# Molecular mechanism of cold acclimation regulating freezing tolerance in *Prunus mume*

**DOI:** 10.1093/plphys/kiag333

**Published:** 2026-06-03

**Authors:** Weixue Liu, Haolin Liu, Xiangbo Liu, Zhoujie Yue, Jiaqi Yan, Liying Wei, Huiying Wang, Pan Zhao, Guangya Bian, Qin Zhang, Yinran Huang, Qixiang Zhang, Tangchun Zheng, Ping Li

**Affiliations:** College of Forestry, College of Landscape and Tourism, Key Laboratory of National Forestry and Grassland Administration on Colorful Tree, Hebei Key Laboratory of Floral Biological Breeding, Hebei Agricultural University, Baoding 071000, China; College of Forestry, College of Landscape and Tourism, Key Laboratory of National Forestry and Grassland Administration on Colorful Tree, Hebei Key Laboratory of Floral Biological Breeding, Hebei Agricultural University, Baoding 071000, China; College of Forestry, College of Landscape and Tourism, Key Laboratory of National Forestry and Grassland Administration on Colorful Tree, Hebei Key Laboratory of Floral Biological Breeding, Hebei Agricultural University, Baoding 071000, China; National Engineering Research Center for Floriculture, School of Landscape Architecture, Beijing Forestry University, Beijing 100083, China; College of Forestry, College of Landscape and Tourism, Key Laboratory of National Forestry and Grassland Administration on Colorful Tree, Hebei Key Laboratory of Floral Biological Breeding, Hebei Agricultural University, Baoding 071000, China; College of Forestry, College of Landscape and Tourism, Key Laboratory of National Forestry and Grassland Administration on Colorful Tree, Hebei Key Laboratory of Floral Biological Breeding, Hebei Agricultural University, Baoding 071000, China; College of Forestry, College of Landscape and Tourism, Key Laboratory of National Forestry and Grassland Administration on Colorful Tree, Hebei Key Laboratory of Floral Biological Breeding, Hebei Agricultural University, Baoding 071000, China; College of Forestry, College of Landscape and Tourism, Key Laboratory of National Forestry and Grassland Administration on Colorful Tree, Hebei Key Laboratory of Floral Biological Breeding, Hebei Agricultural University, Baoding 071000, China; Shijiazhuang Academy of Agriculture and Forestry Sciences, Shijiazhuang 050899, China; College of Forestry, College of Landscape and Tourism, Key Laboratory of National Forestry and Grassland Administration on Colorful Tree, Hebei Key Laboratory of Floral Biological Breeding, Hebei Agricultural University, Baoding 071000, China; College of Forestry, College of Landscape and Tourism, Key Laboratory of National Forestry and Grassland Administration on Colorful Tree, Hebei Key Laboratory of Floral Biological Breeding, Hebei Agricultural University, Baoding 071000, China; National Engineering Research Center for Floriculture, School of Landscape Architecture, Beijing Forestry University, Beijing 100083, China; National Engineering Research Center for Floriculture, School of Landscape Architecture, Beijing Forestry University, Beijing 100083, China; College of Forestry, College of Landscape and Tourism, Key Laboratory of National Forestry and Grassland Administration on Colorful Tree, Hebei Key Laboratory of Floral Biological Breeding, Hebei Agricultural University, Baoding 071000, China

## Abstract

The *Prunus mume* species has undergone a long process of domestication and introduction in China, resulting in marked differences in freezing tolerance among populations across a latitudinal gradient of approximately 1,000 km. However, the introduction and domestication process is time-consuming, constraining the efficiency of species introduction. In this study, *P. mume* samples with the same genetic background spanning over 1,000 km from north to south were selected as experimental materials. The molecular mechanisms underlying cold-acclimation-mediated freeze tolerance in *P. mume* were investigated using multiomics high-throughput sequencing and molecular biology techniques. The cold-acclimated plant material exhibited enhanced freezing tolerance. Cold acclimation substantially enhanced transcriptional reprogramming under cold stress, accompanied by the remodeling of chromatin states in the promoter region. A variety of cis-regulatory elements were identified in the open chromatin regions of cold-acclimated plant materials, including G-box, ABRE, and other stress-responsive elements. The bZIP transcription factor PmGBF1, as a key regulatory node, directly regulates the expression of the cold shock protein gene *PmCSL* by binding to G-box elements to regulate freezing tolerance. This study reveals the molecular mechanism underlying cold acclimation mediated by the PmGBF1-PmCSL pathway in regulating *P. mume* freezing tolerance, providing an epigenetic theoretical basis and genetic resources for cold-acclimation breeding of freezing tolerance in woody plants.

## Introduction

Low temperature is a key abiotic stressor that affects the growth, development, and geographical distribution of plants (Chinnusamy et al. 2007; Zhu 2016) Globally, the majority of tree species are concentrated in the tropical and subtropical regions, whereas the boreal and frigid regions contain relatively few woody perennials, highlighting the strong ecological constraint imposed by low temperature on woody plant survival (Cazzolla Gatti et al. 2022). As sessile organisms, plants cannot actively avoid adverse environmental conditions through spatial migration. Thus, low temperature, particularly freezing stress, becomes a critical ecological factor affecting plant survival (Thomashow 1999; Ninkuu et al. 2023; Huang and Wei 2024). Plants have evolved adaptive strategies such as dormancy and cold acclimation to cope with cold environments (Ritonga and Chen 2020). Dormancy reduces metabolic activity and energy consumption during unfavorable seasons, whereas cold acclimation enhances freezing tolerance after exposure to nonfreezing low temperatures (Knight and Knight 2012; Ding et al. 2019). This process involves a series of physiological and molecular adjustments, including membrane lipid remodeling, osmoprotectant accumulation, antioxidant system activation, and induction of cold-responsive (COR) genes, thereby maintaining cellular homeostasis under low-temperature conditions (Baumann 2017; Kim et al. 2024; Shomo et al. 2024; Zhang et al. 2024a).

The systemic adaptation triggered by cold acclimation is because of epigenetic reprogramming induced by low-temperature signals, relying on the dynamic regulation of chromatin accessibility on a genome-wide scale. In the eukaryotic genome, DNA is highly packaged into nucleosomes, and the physical state of chromatin determines whether cis-regulatory elements are recognized and occupied by transcriptional regulatory factors (Consortium 2012; Kasinathan et al. 2014; Yen et al. 2025). Under the influence of environmental signals, chromatin remodeling factors, structural proteins, and various epigenetic modification systems function in combination to mediate the reversible remodeling of nucleosome positioning, chromatin compactness, and accessibility of regulatory elements, thereby providing a structural basis for the activation of stress response-related genes (Song et al. 2021; Wang et al. 2023). Under low-temperature stress conditions, chromatin regions near the stress response-related genes usually undergo transformation from a relatively compact conformation to a more open state (Wang et al. 2021; He et al. 2025). The chromatin in the open conformation is more accessible to transcriptional regulatory factors and more susceptible to fine regulation by various epigenetic mechanisms such as DNA methylation, chromatin remodeling, and histone modification. These modifications are highly reversible and can directly regulate gene transcription (Ding et al. 2019; Gehring 2019; Li et al. 2019; Zhao et al. 2019; Zou et al. 2025). These findings indicate that chromatin remodeling plays a substantial role in the low-temperature-induced transcriptional regulation of herbaceous and woody plants. However, the specific regulatory mechanism underlying this process in woody plants remains unclear. Meanwhile, the low-temperature signal can induce an epigenetic transition of chromatin from an inhibitory state to a permissive state, manifested as the remodeling of inhibitory marks and the establishment of activating conformations, forming a stress-induced epigenetic switch (Park et al. 2018; Song et al. 2024). In addition to the chromatin state changes at the linear genomic level, environmental stress can further trigger higher-level chromatin structural reorganizations, altering the spatial interaction patterns between distant regulatory elements and the promoter regions of target genes, achieving coordinated regulation of stress-responsive genes (Huang et al. 2023; Yen et al. 2025). This dynamic change in chromatin conformation is highly coupled with transcriptional activity, highlighting the functional significance of spatial organization of chromatin in response to environmental signals (Zhang et al. 2024a, 2024b).

In the context of chromatin remodeling mediated by low-temperature signals, transcription factors (TFs) integrate and transmit regulatory information from the epigenetic to transcriptional level by binding to cis-regulatory elements related to stress response genes. The bZIP family, as a unique and functionally diverse TF family in plants, has attracted considerable attention because of its crucial roles in light signals, abiotic stress, and hormone signaling pathways (Hsieh et al. 2012; Hartmann et al. 2015; Zong et al. 2016). Members of this family recognize ACGT core sequences (G-box and ABRE) and bind to target genes, mediating the regulation of gene expression under stress conditions (Ezer et al. 2017). Previous studies have shown that multiple bZIP TFs are directly involved in the response to low-temperature stress and the regulation of cold tolerance in plants. BZIP73 participates in regulating the cold response-related gene network under low-temperature conditions and plays a crucial role in the formation of cold tolerance during the reproductive stage (Liu et al. 2019). Meanwhile, bZIP68 inhibits COR gene expression under low-temperature stress, thus exerting a negative regulatory role on cold tolerance (Li et al.  2022b). Furthermore, some bZIP TFs participate in the cold adaptation process by regulating antioxidant and secondary metabolic pathways. For instance, AcePosF21 enhances the antioxidant capacity of cells by activating genes involved in ascorbic acid biosynthesis, whereas SlAREB1 mediates the cold-induced biosynthesis of anthocyanins, helping maintain oxidative homeostasis under cold stress conditions (Liu et al. 2023; Xu et al. 2023). The functional activity of bZIP TFs often relies on higher-level regulatory methods at the regulatory mechanism level. For instance, the interaction between *DgbZIP3* and *DgbZIP2* synergistically activates the expression of antioxidant-related genes, and variable splicing of *CiFD* enables an integrated response to low temperatures and other adverse environmental signals (Bai et al. 2022; Ye et al. 2023). These studies collectively demonstrate that bZIP TFs play a crucial hub-like regulatory role in the transmission of low-temperature signals, changes in chromatin state, and transcriptional reprogramming. However, at present, a lack of systematic understanding persists regarding the role of bZIP TFs in the cold acclimation process of perennial woody plants. An indepth analysis of the regulatory function and mechanism of bZIP TFs provides an important theoretical basis for elucidating the molecular regulatory network of cold responses in perennial woody plants.

*Prunus mume*, a species in the genus *Prunus* of the family Rosaceae, is a traditional flower in China, holding significant economic, ornamental, and cultural value (Wu et al. 2022; Huang et al. 2025). The Yangtze River Basin is regarded as the main origin of the diversity of Mei varieties. The significant differences in latitude gradients and winter cold intensity across different cultivation areas lead to obvious regional differentiation in the cold-acclimation ability and cold tolerance levels of Mei populations (Zhang et al. 2012, 2018). After nearly 70 years of systematic breeding, regional introduction, and domestication, the cold resistance of certain plum varieties has been significantly enhanced, enabling them to survive safely in open fields and flower normally in northern regions such as Beijing (BJ). However, the molecular basis of cold hardiness formation and regulation during the cold acclimation process of *P. mume* requires further study. Therefore, analyzing the molecular basis of cold hardiness formation and regulation during cold acclimation is crucial for understanding the cold adaptation mechanism of woody plants and for breeding cold-resistant varieties. Based on homology analysis, G-box Binding Factor 1 (*PmGBF1*) is identified as the previously reported *bZIP44* (Li et al. 2022a, 2022b). In this study, based on the *P. mume* materials from BJ and Wuhan (WH), combined with multiomics analysis and cold acclimation treatment, we systematically analyze the regulatory characteristics of PmGBF1 under different low-temperature adaptation backgrounds, and further propose the molecular mechanism by which it directly regulates the cold shock protein (CSP) encoding gene *PmCSL* to participate in the formation of cold tolerance. This study aimed to decipher the coupling mechanism of chromatin remodeling and transcriptional regulation during cold acclimation in *P. mume*, clarify the regulatory function of PmGBF1 and its downstream target genes, and reveal the core molecular pathway underlying the formation of freezing tolerance in *P. mume*.

## Results

### Quantitative evaluation of freezing resistance of *P. mume* based on the median lethal temperature (LT_50_)

The annual branches, as the main plant tissue of *P. mume*, were subjected to frost-damage treatment under controlled temperature conditions on plant materials from the BJ and WH regions. As the processing temperature gradually decreased, the relative ion leakage rates of the 2 plant materials showed a continuous upward trend, indicating that the degree of damage to the cell membrane system under low-temperature conditions intensified. Within the temperature range of 0 to −10 °C, the increase in ion leakage rate was gradual, indicating that the overall stability of the cell membrane remained high. When the temperature decreased further to −15 °C or below, the ion leakage rate increased sharply, indicating that the cell membrane integrity was compromised and a large amount of electrolyte leaked from the cell. The median lethal temperature (LT_50_) of the 2 materials was calculated by fitting a logistic curve to the relationship between ion leakage rate and temperature (Fig. S1). Among them, the LT_50_ of plant materials from the WH region was −10.18 °C, while that of plant materials from the BJ region was −14.35 °C. As a key physiological indicator of plant cold resistance, the lower the LT_50_ value, the lower the low-temperature limit the tissue can tolerate before irreversible damage occurs, and the stronger the cold resistance. Compared with plant materials from the WH region, the LT_50_ of plant materials from the BJ region was significantly reduced by approximately 4.17 °C, clearly indicating that the plant materials from the BJ region had higher cell membrane stability and a stronger physiological basis for cold resistance under low temperature conditions.

### Long-term geographical differences affect the expression pattern of *P. mume* response to low temperature

**Figure 1:**
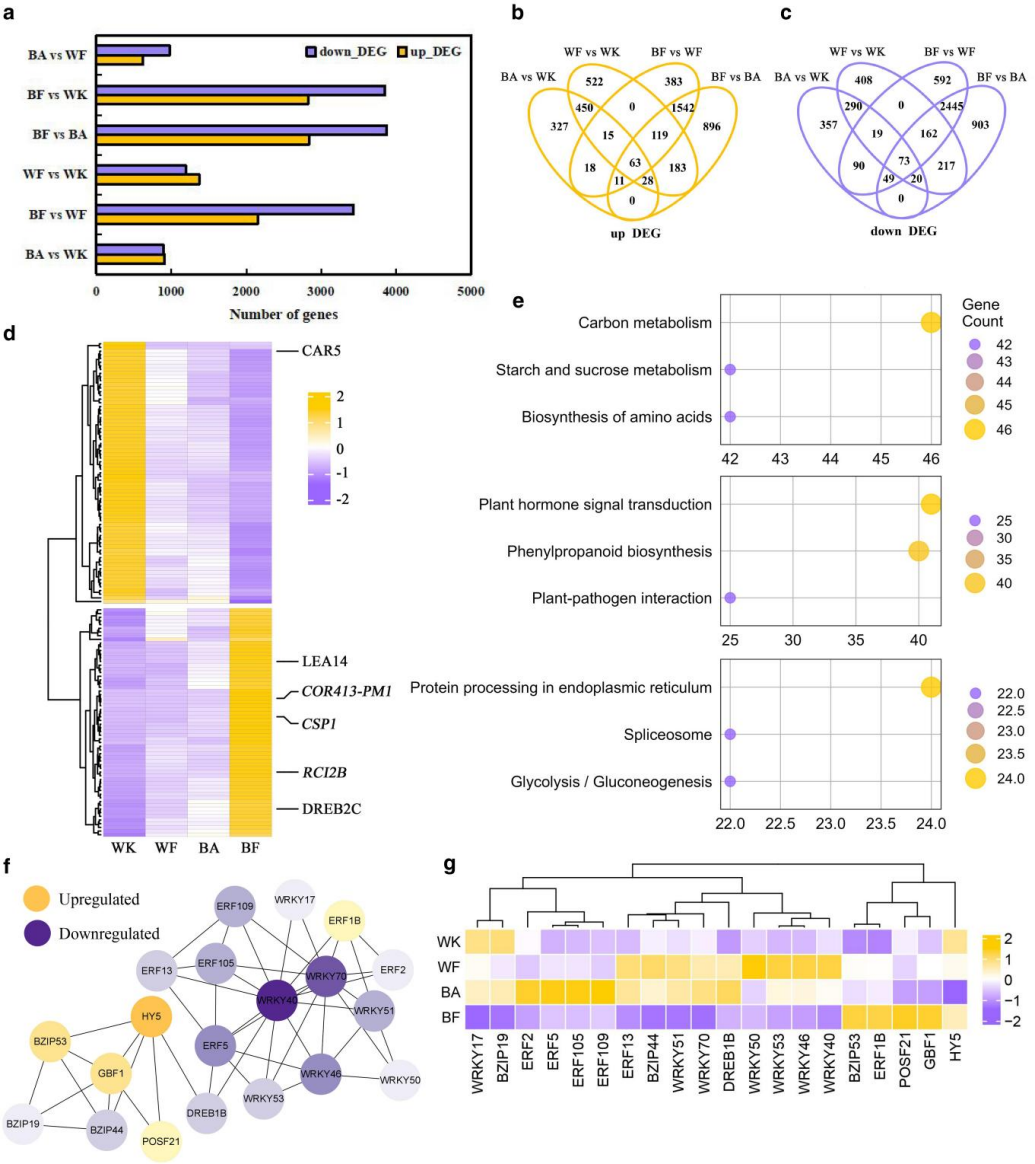
DEGs and functional enrichment analyses across treatments in *P. mume*. a) Numbers of DEGs identified in each pairwise comparison among the 4 treatment groups. b) Venn diagram showing the overlap of upregulated DEGs among comparisons. c) Venn diagram showing the overlap of downregulated DEGs among comparisons. d) Heatmap of expression profiles for the 136 DEGs shared across treatments. Each row represents one gene. e) KEGG enrichment analysis of shared DEGs from the comparisons BF vs. BA and BF vs. WF, showing the top 9 significantly enriched pathways. f) TF interaction network constructed from DEGs in the BF vs. BA comparison, highlighting the top 20 nodes ranked by connectivity. g) Heatmap of TF expression profiles in the BF vs. BA comparison. Abbreviations: WK, WH control WF, freezing treatment in WH; BA, BJ control; BF, freezing treatment in BJ.

The gene expression profile showed high correlation among the 3 biological replicates, indicating good repeatability and reliability (Fig. S2). In plant materials from the WH region, a total of 1,380 upregulated and 1,189 downregulated differentially expressed genes (DEGs) were detected under short-term freezing stress treatment (WF, −4 °C, 4 h), indicating that short-term freezing stress induced significant, relatively balanced transcriptional remodeling. In contrast, the plant materials from the BJ region demonstrated a more extensive transcriptional response to the same freezing treatment under the background of cold acclimation. A total of 2,842 upregulated DEGs and 3,869 downregulated DEGs were identified, with downregulated genes accounting for a larger proportion, suggesting that long-term cold acclimation was accompanied by a wider spectrum of transcriptional reprogramming (Fig. 1a). A total of 136 shared DEGs were identified in the 4 treatment combinations, including 63 upregulated and 73 downregulated genes (Table S2; Fig. 1b and 1c). These genes may show differential expression across all low-temperature-related treatments, suggesting that they might constitute the core regulatory module of the low-temperature response. Hierarchical clustering analysis of its expression pattern revealed that it could be divided into 2 main expression clusters. One cluster was highly expressed overall in plant materials from the BJ region freezing treatment group and contained 5 genes or proteins related to freezing stress (Fig. 1d). In comparing the response of *P. mume* to freezing stress in different regions, a total of 1,542 upregulated DEGs and 2,445 downregulated DEGs were identified. A total of 658 genes were annotated into 141 pathways. Multiple pathways showed significant enrichment of genes (Table S3; Fig. 1e), indicating that the regulatory process of long-term cold response resulted from the combined action of multiple pathways (Table S3; Fig. 1e), indicating that the hormone-mediated regulatory process played a key role in the cold response. In the 4 comparison groups, 89, 108, 59, and 102 TFs were identified as differentially expressed in the same region across treatments and in different regions within the same treatment. These TFs were primarily from the ZF-HD, AP2/ERF, WRKY, HSF, and bZIP families (Table S4; Fig. S3). Protein–protein interaction network analysis revealed that there were dense interactions among the encoded proteins of these core TFs (Figs 1f; Fig. S4). Further cluster analysis based on the expression levels of TFs in the plant materials from the BJ region revealed that members of the WRKY, ERF, and bZIP families showed significant differential expressions (Fig. 1g), suggesting that they might have been involved in the response to low temperature stress through synergistic regulation.

### Long-term geographical differences affect the chromatin remodeling of *P. mume* response to low temperature

**Figure 2:**
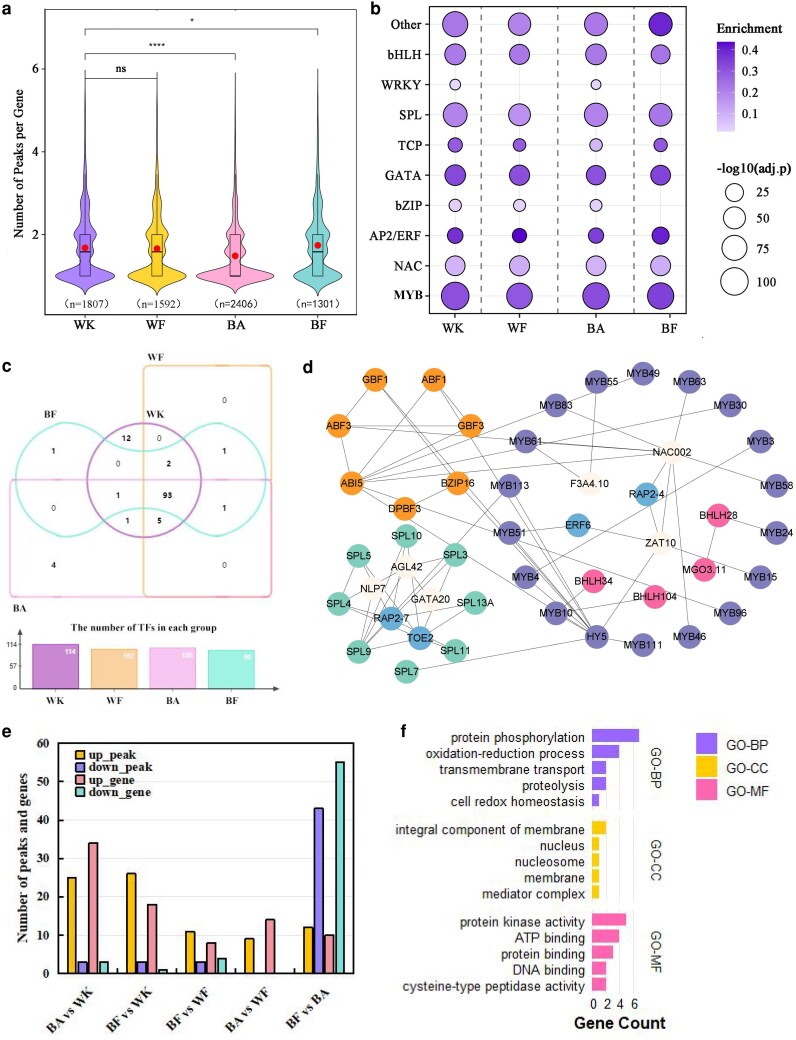
Chromatin accessibility changes and regulatory factor identification. a) Number and distribution of genes corresponding to the accessibility peaks of the WK, WF, BA, and BF groups. The solid dots represent the average values. The significant differences between groups were expressed as *P < 0.05, ****P < 0.0001, and ns indicated no significant difference. b) Bubble plots of the TF family motifs enriched in the 4 groups of samples. The shading intensity indicates the enrichment degree (TP/pos), and the size of the bubbles represents the significance level (−log10 (adj. *P*). c) Four groups share the TFs' Venn diagram. d) Interaction network diagrams of TFs predicted peaks for the WK, WF, BA, and BF groups. Nodes represent different TFs. e) Number of different peaks in each comparison group and the corresponding number of annotated genes. f) Bar chart of GO enrichment analysis results of differentially expressed peaks annotated genes. Abbreviations: WK, WH control WF, freezing treatment in WH; BA, BJ control; BF, freezing treatment in BJ.

High-quality chromatin accessibility data were obtained for all 12 samples, with each sample yielding more than 80 million uniquely aligned, valid reads (the data volume per sample ranged from 124 to 244 GB). The sequencing quality was stable, with Q20 and Q30 exceeding 94% and 87%, respectively. The transcription start site (TSS) signal showed clear enrichment upstream and downstream of the TSS (Fig. S5), indicating that the library quality met the requirements for open chromatin analysis. Genomic annotation showed that the peaks were mainly distributed in gene promoters and distal intergenic regions (Fig. S6). Correlation analysis showed strong correlation among the 3 biological replicates, indicating good repeatability and reliability (Fig. S7). The peak annotation results across different treatment groups showed that plant materials from the BJ region had a higher number of accessible regions and their associated genes (Fig. 2a), suggesting that cold acclimation may broaden the potential for gene regulation. The number of identified significant motifs was as follows: for the WH control and freeze treatment groups, 166 and 154, respectively; for the BJ control and freeze treatment groups, 159 and 152, respectively. These corresponded to the prediction of 114, 102, 105, and 99 TFs (Tables S5 to S8). There were 93 shared TFs across the 4 groups. Notably, compared with the WH materials, the motif enrichment levels of the bHLH, bZIP, and GATA transcription factor families in the BJ materials were generally higher (Fig. 2b and 2c), suggesting that their transcriptional regulatory characteristics might be influenced by cold acclimation. Further analysis of the TFs revealed that 47 predicted TFs formed an interaction network, among which MYB, bZIP, and members of the SQUAMOSA promoter-binding protein family accounted for a significant proportion (Fig. 2d). The differential accessibility analysis indicated a higher number of differential peaks and their associated genes in the BJ plant materials (Fig. 2e), suggesting that cold-acclimated tissues underwent more extensive chromatin accessibility remodeling when subjected to similar cryotherapy treatment. The analysis of genes associated with all differential peaks indicated their primary involvement in key biological processes, including signal transduction, metabolic regulation, stress response, and DNA repair (Fig. 2f; Tables S9 and S10). Notably, enrichment of signal transduction pathways indicated that cold acclimation enhanced the ability of tissues to sense and rapidly transmit signals of low-temperature stress. In contrast, activation of stress response and DNA repair pathways suggested that acclimation initiated protective mechanisms. These processes functioned in combination to improve the cold tolerance of plants.

### Epigenetic regulatory factors affecting the freezing tolerance of *P. mume* under cold acclimation

**Figure 3:**
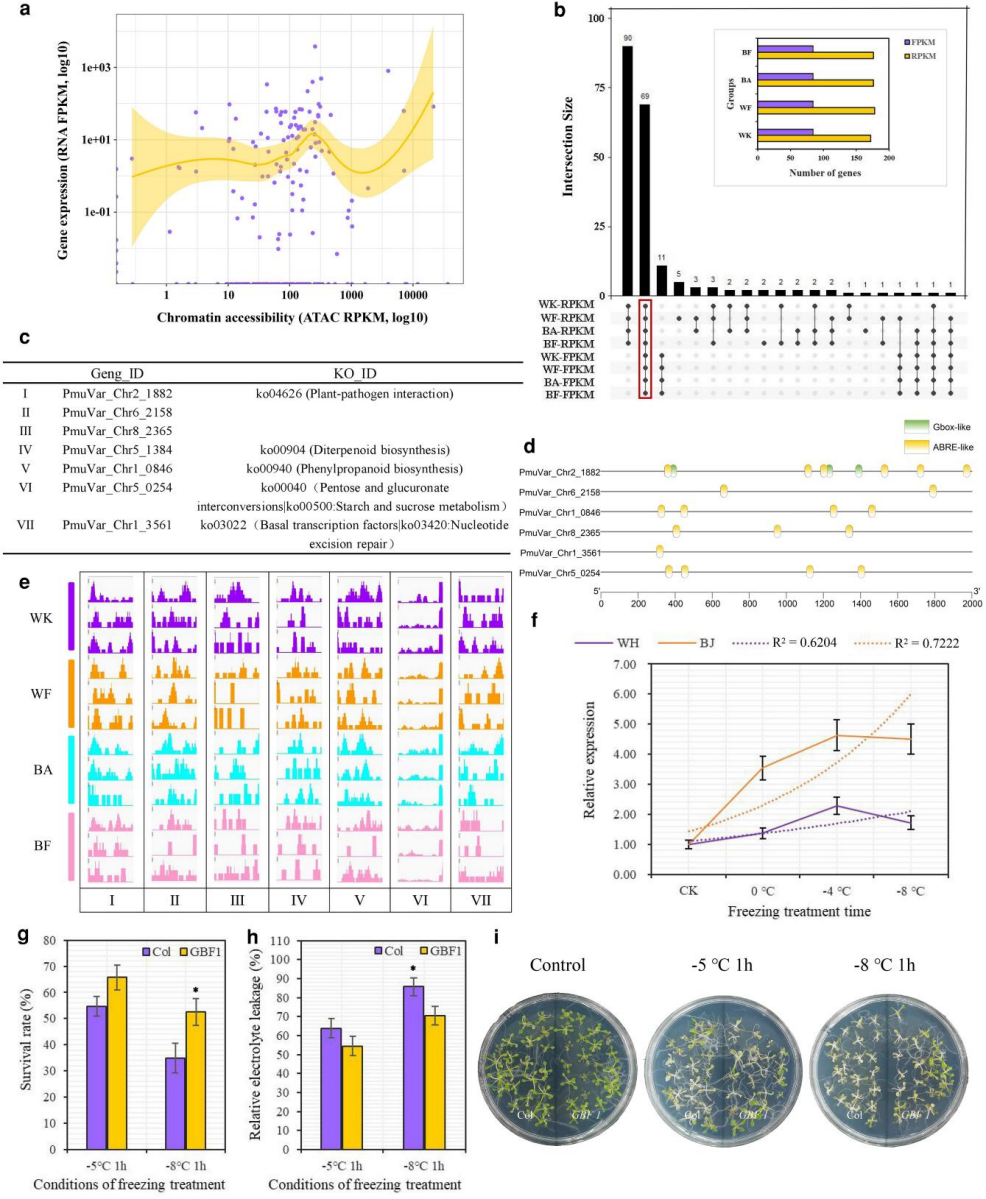
Integrated multiomics analysis identifies and validates PmGBF1 as a key TF enhancing freezing tolerance. a) Trend of the correlation between chromatin accessibility and gene expression levels. b) Intersection analysis of genes corresponding to medium and high chromatin open regions and genes with high RNA-seq expression. c) Seven cold-resistance genes among the 69 intersection genes and their KEGG pathways. d) Analysis of G-box and ABRE-like cis-elements in the 7 genes. e) Chromatin open peak maps of the promoter regions of the 7 genes. f) Relative expression level of qRT-PCR gene. g, h) Survival rates and ion permeability of wild-type (Col) and PmGBF1-overexpressing (PmGBF1-OE) plants after freezing treatment. Data are presented as mean ± standard deviation (*n* = 3). * indicates significant differences between Col and PmGBF1-OE plants under the same treatment conditions (chi-square test, *P < 0.05). i) Cold treatment phenotypes of wild-type (Col) and overexpressed gene plants.

The chromatin accessibility of each gene in the 2 kb region upstream of the TSS was calculated from read counts and standardized as reads per kilobase per million (RPKM) (Tables S11 and S12). Statistics on genes with different openness levels showed that genes in promoter regions with high and medium openness accounted for more than 70% across all 4 treatment groups, indicating that the regulatory regions of many genes maintain a relatively open chromatin state. Further analysis was conducted on the open level of the promoter region, and the fragments per kilobase per million (FPKM) value of the gene, and the correlation between RPKM and FPKM was evaluated. Chromatin accessibility in the promoter region showed a weak but positive correlation with gene expression, indicating that increased chromatin openness was usually associated with increased transcriptional activity. The genome structure of woody plants is complex, containing numerous repetitive and diverse regulatory elements, leading to multilevel regulation of gene expression. Although chromatin openness can enhance transcriptional activity, changes in gene expression may be influenced by local regulation and temporal and spatial specificity, resulting in a weak positive correlation between openness and expression (Fig. 3a). To identify key genes involved in the response to low temperatures, genes in the “High” and “Intermediate” regions were selected, and the intersection with the highly expressed genes identified by RNA-seq was examined. A total of 69 genes were identified that simultaneously exhibited both high expression and high chromatin accessibility (Fig. 3b; Table S13). Analysis of the cis-acting elements in their promoter regions revealed that these genes were significantly enriched for typical regulatory elements, including the CAAT box, MYB, G-box, and ABRE-like elements. The functional annotation results indicated that the 7 representative genes were involved in 5 metabolic or signaling pathways related to cold response (Fig. 3c), and their promoter regions generally contained G-box and ABRE-like elements recognized by bZIP-like TFs (Fig. 3d). Meanwhile, all 7 genes exhibited significantly enhanced chromatin openness during cold acclimation (Fig. 3e), suggesting that they were relatively highly transcribed under low-temperature induction, conducive to TF binding and activation of downstream cold response pathways.

### PmGBF1 mediates cold acclimation-induced epigenetic and transcriptional reprogramming to confer freezing tolerance

Combining expression patterns with changes in chromatin structure, the expression levels of *PmGBF1* significantly increased after cold acclimation treatment (Fig. 1f and 1g), accompanied by enhanced chromatin openness near their target genes. Moreover, these TFs showed clear interaction networks in both ATAC-seq and expression matrices (Fig. 2d). In addition, other gene promoter regions associated with cold acclimation, with both high expression and high openness, were enriched in cis-acting elements to which bZIP could bind, suggesting that *PmGBF1* may play a key role in the regulatory network induced by cold acclimation. Meanwhile, the protein as a whole exhibited strong hydrophilicity (Fig. S8), consistent with its structural characteristics as a nuclear-localized TF (Fig. S8). Under freezing treatment, the expression of *PmGBF1* was significantly increased in both types of *P. mume*. Among them, the expression level was the highest under −4 °C treatment, and the expression level of BJ *P. mume* was consistently higher than that of WH *P. mume* (Fig. 3g). Correspondingly, the PmGBF1-overexpressing *Arabidopsis* plants exhibited stronger cold resistance: before cold acclimation, their survival rate at −5 °C was already higher than that of the wild-type; after cold acclimation, the survival rate of the transgenic plants at −8 °C significantly increased, and the ion permeability was significantly reduced, indicating that the cell membrane integrity was better maintained (Fig. 3h and 3i). Collectively, these results indicated that PmGBF1 was a positive regulator of cold stress and could significantly enhance plant cold resistance, especially under the background of cold acclimation.

### Genome-wide binding and target gene regulation by PmGBF1 affect the freezing tolerance of *P. mume* under cold acclimation

**Figure 4:**
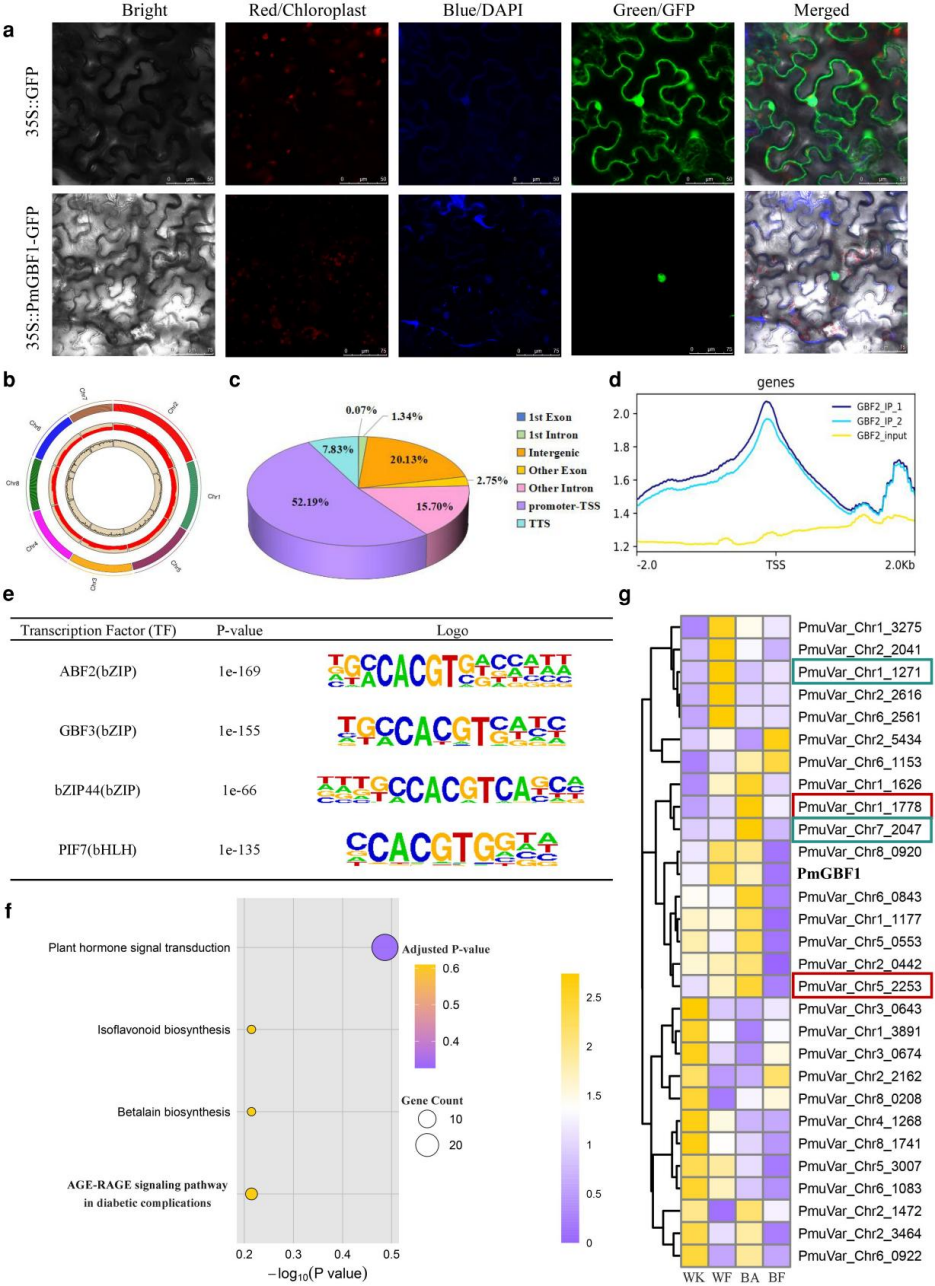
Identification and functional characterization of PmGBF1 binding sites by DAP-seq analysis. a) Subcellular localization of PmGBF1. b) Distribution signal map of binding peaks on each chromosome throughout the genome. c) Distribution of PmGBF1 binding sites on the genome. d) Distribution frequency of peaks on both sides of the TSS. e) PmGBF1 binding motif. f) KEGG enrichment analysis of genes with associated peaks located in the promoter–TSS region and in the top 2,000 of enrichment significance. g) Clustering heatmap analysis of 29 genes in the plant hormone signal transduction pathway.

The subcellular localization results showed that in the leaf epidermal cells carrying the green fluorescent protein (GFP)-PmGBF1 recombinant plasmid, the GFP fluorescence signal was specifically enriched in the cell nucleus (Fig. 4a), indicating that the PmGBF1 protein was localized in the cell nucleus. Subsequently, the DAP-seq data showed high consistency between the 2 biological replicates, indicating reliable results (Fig. S9). A total of 27,122 PmGBF1 binding peaks were identified in the *P. mume*. These peaks were distributed across all 8 chromosomes and were predominantly enriched in promoter–TSS regions (52.19%), with additional peaks located in intergenic (20.13%) and intronic regions (15.7%) (Fig. 4b to 4d), suggesting that PmGBF1 mainly functions as a transcriptional regulator acting on gene promoters. Motif analysis revealed that PmGBF1 preferentially binds to cis-elements containing the ACGT core sequence, including the classical G-box (CACGTG) and ABRE-like motifs, characteristic recognition motifs for bZIP TFs (Fig. 4e). Based on the genomic regions associated with PmGBF1 binding peaks, 15,339 peak-associated genes were annotated. Among these, genes with binding peaks in promoter–TSS regions were ranked by enrichment significance, and the top 2,000 genes were selected for functional analysis. These genes were significantly enriched in the plant hormone signal transduction pathway (Fig. 4f). Further clustering analysis revealed that several stress-related hormone signaling genes, including *PYL9* and *PYL4* in the abscisic acid signaling pathway, as well as *EIN2* and *MYC2* in the ethylene and jasmonic acid signaling pathways, showed relatively high expression levels under low-temperature treatment and exhibited expression patterns similar to that of PmGBF1, suggesting a potential regulatory relationship between PmGBF1 and these downstream genes (Figure 4g; Table S14).

### Long-term geographical adaptation enables PmGBF1-mediated regulation of *PmCSL* in response to low temperature

**Figure 5:**
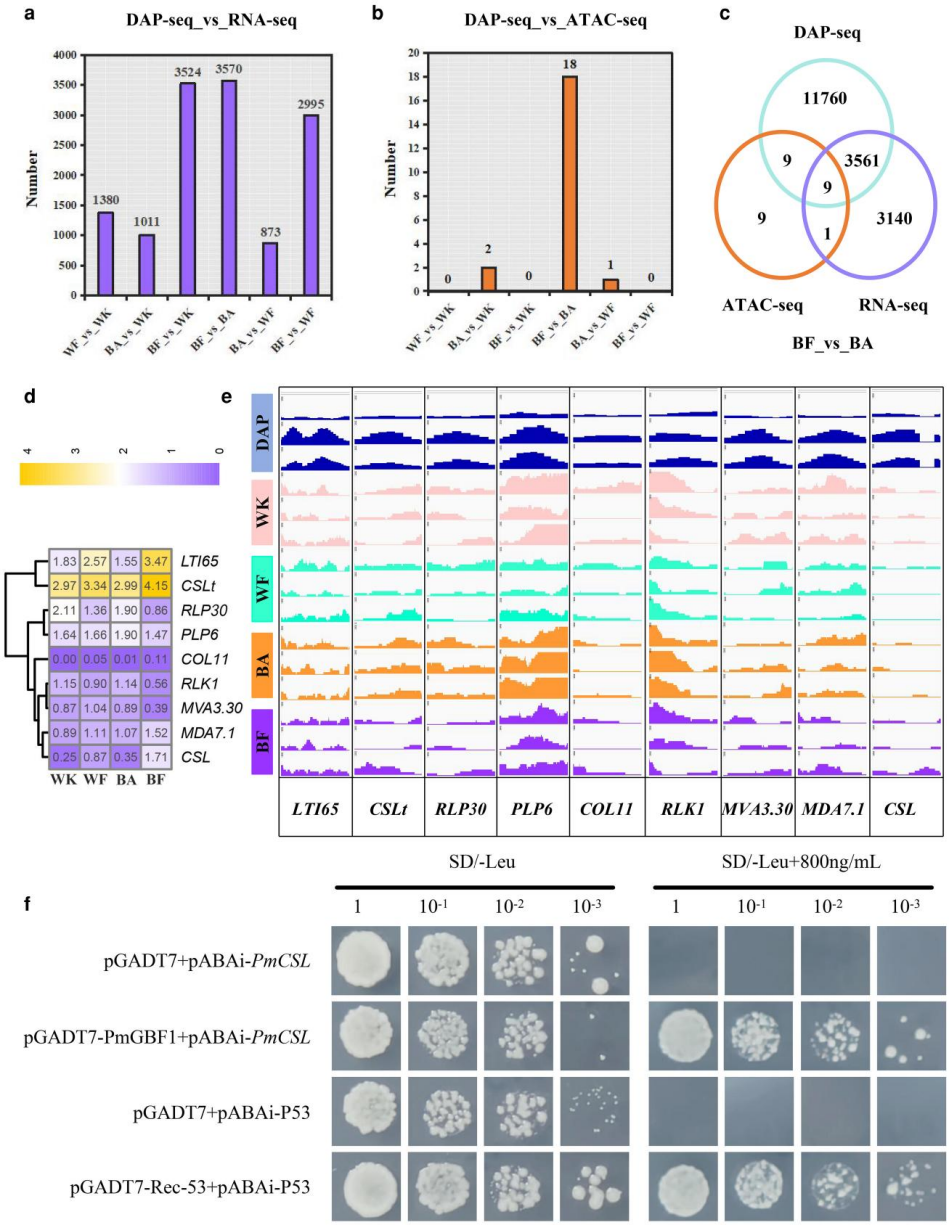
Identification of potential direct target genes of PmGBF1 and verification of its regulatory effect on *PmCSL*. a) Intersection of the target gene annotated at the PmGBF1 binding site and the DEG. b) Intersection of the target gene annotated at the PmGBF1 binding site and the gene annotated in the chromatin open region. c) Intersection of PmGBF1 target genes, chromatin-open region genes, and DEGs. d) Heatmap cluster analysis of gene expression levels for intersection genes. e) Peaks of intersection genes at PmGBF1 binding sites and in chromatin-open regions. f) Yeast single-hybrid verification.

To comprehensively analyze the regulatory mechanism of PmGBF1, a joint analysis of multiomics data was conducted. First, potential target genes of PmGBF1 that possessed both binding sites and expression responses were screened out. The results showed that the number of such target genes in plants subjected to cold treatment in BJ was higher than that in other groups (Fig. 5a). Further, high-confidence binding sites within open chromatin regions were obtained and annotated to the nearest coding genes. The results showed that the BJ plant materials had the largest number of annotated genes, and that PmGBF1 binding to open chromatin regions in BJ P. mume was more extensive during long-term geographical adaptation. These results indicated that the chromatin regulatory landscape of BJ *P. mume* might have undergone adaptive remodeling, providing a structural basis for the more efficient binding and regulation of downstream target genes by PmGBF1 (Fig. 5b). Subsequently, highly reliable potential direct regulatory genes of PmGBF1 were obtained through intersection analysis. The results showed that the number of intersection genes in the BJ freezing treatment group was significantly higher than in other treatment groups, and PmGBF1 activated the downstream target gene network of *P. mume* on a larger scale under acute low-temperature stress after cold acclimation (Fig. 5c). The expression levels of 9 genes were analyzed by clustering, and 6 of them showed a general upward trend (Fig. 5d). These genes were significantly expressed under cold acclimation. At the same time, DNA binding peaks and chromatin openness peaks were observed at the binding sites of the 9 genes (Fig. 5e). Among these, the expression levels of *PmCSL* and its tandem duplicate, *PmCSLt*, significantly increased under BJ freezing treatment. The DAP-seq results indicated that compared to *PmCSLt*, GBF1 exhibited a stronger binding signal in the promoter region of *PmCSL*. Therefore, further studies on *PmCSL* were conducted. Domain analysis indicated that the PmCSL protein contains a dehydrin conserved domain. Phylogenetic analysis showed that PmCSL clusters with homologous proteins from other *Prunus* species, with high conservation observed within this lineage (Fig. S10). The chromatin openness of *PmCSL* increased under BJ cryopreservation. The results of the yeast one-hybrid experiment showed that the bait strain containing the *PmCSL* element was completely inhibited in its growth on the SD/-Ura plate at 800 ng/mL AbA. The pAbAi-*PmCSL* bait, when combined with AD-PmGBF1, could grow normally on the SD/-Leu plate at 800 ng/mL AbA, while the corresponding mutant bait combinations failed to grow (Fig. 5f). These results demonstrated that PmGBF1 specifically bound to the cis-acting elements within the *PmCSL* promoter.

## Discussion

*P. mume* is one of the well-known traditional flowers in China and an important garden tree species, which has ornamental, ecological, and cultural values. As a typical perennial woody plant, its cold resistance does not stem from a plastic response induced by short-term low-temperature stimulation, but rather from a cold acclimation state gradually established and maintained under repeated and long-term low-temperature conditions (Zhang and Sun 2019; Guo et al. 2024b). In this study, the freezing tolerance of “Zaohua Lve” Mei materials was quantitatively evaluated through the LT_50_ and ion permeability. The results showed that the materials formed under multiyear cold-acclimation conditions exhibited substantially reduced LT_50_ values and lower ion permeability during freezing treatment, indicating enhanced cell membrane integrity and overall freezing tolerance. Similar cold-tolerance phenotypes, including “low LT_50_ and low ion permeability,” have been confirmed in a variety of woody plants (Artlip et al. 2016; Li et al. 2020; Larter et al. 2026). Unlike studies that mainly rely on short-term cryogenic treatment to reveal transient response differences, the notable differentiation in LT_50_ and ion permeability observed in this study is more likely to reflect a long-term, persistent physiological state of cold acclimation. Notably, LT_50_ does not reflect a single physiological process, but rather the combined output of multiple cold resistance mechanisms such as membrane lipid remodeling, protective molecule accumulation, and transcriptional and translational homeostasis (Shi et al. 2018; Ding et al. 2019). This stable physiological difference provides a key basis for subsequent analysis of the regulatory mechanisms related to cold acclimation.

The transcriptional response differences between the WH and the BJ material under freezing stress demonstrated regulatory differentiation beyond the range of gene expression and were closely related to chromatin accessibility remodeling. The WH materials that had not undergone cold acclimation induced only a moderate number of DEGs with balanced upregulation and downregulation after short-term freezing treatment, consistent with acute stress transcriptional responses in various nonadaptive tissues. In contrast, the BJ materials that underwent long-term cold acclimation showed significantly expanded transcriptional reprogramming under the same freezing treatment, mainly downregulating genes, indicating that cold acclimation prioritized stress tolerance by inhibiting growth and metabolic processes. Similar large-scale transcriptional remodeling had been reported in *Solanum tuberosum* under drought and salt stress, and in *Camellia sinensis* under cold stress (Wang et al. 2021; Wen et al. 2025). Notably, only a few genes exhibited consistent differential expression in all low-temperature comparisons, suggesting that the freeze response relied on plasticity to regulate recombination rather than fixed modules, consistent with the evolutionary evidence that the absence of the WRKY34 promoter in *Solanum lycopersicum* led to decreased chromatin access and loss of cold tolerance (Guo et al. 2024a, 2024b). Corresponding to the transcriptional changes, the stronger cold response in the BJ material was accompanied by broader chromatin accessibility remodeling. ATAC-seq showed that BA and BF have more cold-induced open chromatin regions, mainly enriched in the promoter–TSS region, suggesting that cold acclimation substantially expanded the regulatory potential. A similar promoter-centered type accessibility regulation had been confirmed in *Rosa rugosa*, *Vitis amurensis*, and *C. sinensis* (Ren et al. 2021; Wang et al. 2021; Zhang et al. 2025), where stress response TFs preferentially act on newly opened chromatin to drive downstream gene expression. Studies on *Oryza sativa* OSNMCP1-OSSWI3C and *Malus pumila* MDRAD5B-MDLHP1 show that chromatin accessibility was actively regulated by structural proteins and epigenetic modifications (Yang et al. 2020; Song et al. 2024). Overall, when woody perennial plants respond to sudden cold stress, they rely more on the chromatin accessibility state established during the cold acclimation stage to achieve efficient and coordinated transcriptional reprogramming.

In this study, PmGBF1 was identified as an integrated transcriptional regulatory factor in the response to cold stress, and its function is reflected in the regulation of the overall coordination ability of the cold response transcriptional program. Under cold acclimation and freezing treatment conditions, PmGBF1 mainly bound to the bZIP cis-acting element adjacent to the promoter. Its potential target genes are widely involved in core cold-stress processes such as membrane stability, osmotic regulation, and maintenance of cell homeostasis, suggesting that PmGBF1 enhances the systematic nature and stability of the cold response by synergistically activating multiple target genes. Previous studies have shown that bZIP TFs play a significant role in plant cold adaptation. For instance, *OsbZIP72* (Gu et al. 2024) and *OsbZIP73* (Liu et al. 2019) in *O. sativa* are involved in cold tolerance regulation during the seedling and reproductive stages, respectively, and the natural variation of *OsbZIP73* is related to the evolution of *O. sativa* subsp. japonica's adaptation to cold climates (Liu et al. 2018). *OsbZIP83* enhances low-temperature buffering capacity by synergistically regulating melatonin metabolism with *OsCOMT15* (Li et al.  2025b), whereas *Z. mays ZmbZIP68* affects the intensity of the cold stress response (Li et al. 2022a, 2022b), reflecting the species dependence of the bZIP family's functions. Compared with herbaceous plants, perennial woody plants typically experience seasonal temperature fluctuations over a long period. Therefore, they require a more stable and coordinated regulatory system to cope with repeated low-temperature stress. In this context, the regulatory activities of bZIP TFs in woody plants may be more dependent on chromatin-level regulation and the coordinated transcriptional activation of multiple downstream genes, rather than the common rapid but relatively short response mechanisms observed in herbaceous plants. In perennial woody plants, the bZIP factor is associated with energy metabolism and antioxidant processes (Liu et al. 2023; Zhao et al. 2026). Based on this, this study further indicates that the transcriptional activation ability of PmGBF1 depended on the enhanced chromatin accessibility induced by low temperature, and its function is established on the structural basis that cis-elements can be effectively occupied, thus providing regulatory space for multigene synchronous responses. The results of functional verification showed that the continuous activation of PmGBF1 can stably increase the survival rate of plants under cold treatment stress, supporting its role as a key coordinating factor in the cold response regulatory network, and suggesting that the bZIP TF can participate in the construction of a transcriptional regulatory environment conducive to rapid response under the background of cold acclimation.

Under low temperature stress, the bZIP TF does not merely regulate individual cold response genes, rather it regulates the downstream defense, growth inhibition, and homeostasis maintenance processes in a coordinated manner by activating hormone signaling-related modules (Guiltinan et al. 1990; Cai et al. 2018; Li et al. 2022a, 2022b). This study further revealed, through systematic analysis of the PmGBF1 target gene by DAP-seq, that its regulatory network was functionally significantly enriched in the plant hormone signaling pathway, with a concentration at the key regulatory nodes of ABA, ethylene, and jasmonic acid signaling. This result is highly consistent with the previous understanding of the bZIP TF as a hormone signal integrator (Liu et al. 2019; Bai et al. 2022). Notably, in this target gene framework dominated by hormone signaling pathways, this study, through joint analysis, further identified related genes such as E3 ubiquitin ligase (MDA7.1) (Geng et al. 2025), phospholipase (PLP6) (Davidson et al. 2025), dehydrating protein (LTI65) (Gao et al. 2021), and CSP as direct regulatory target genes of PmGBF1. Furthermore, this study validated the direct regulatory relationship involving the CSP gene *PmCSL*. The results of yeast single-hybridization confirmed that PmGBF1 specifically binds to the cis-acting element in the PmCSL promoter, providing evidence for its direct transcriptional regulation of CSP-related genes. Studies have shown that the plant CSP family is highly conserved in evolution, mainly serving as RNA chaperones to maintain RNA homeostasis and post-transcriptional regulation under low temperature conditions (Karlson and Imai 2003), and playing a key role in the low-temperature adaptation and environmental response of *Triticum aestivum*, *Arabidopsis thaliana*, Solanaceae plants, etc. (Sasaki et al. 2007; Li et al. 2025a, 2025b; Wang et al. 2025).

**Figure 6:**
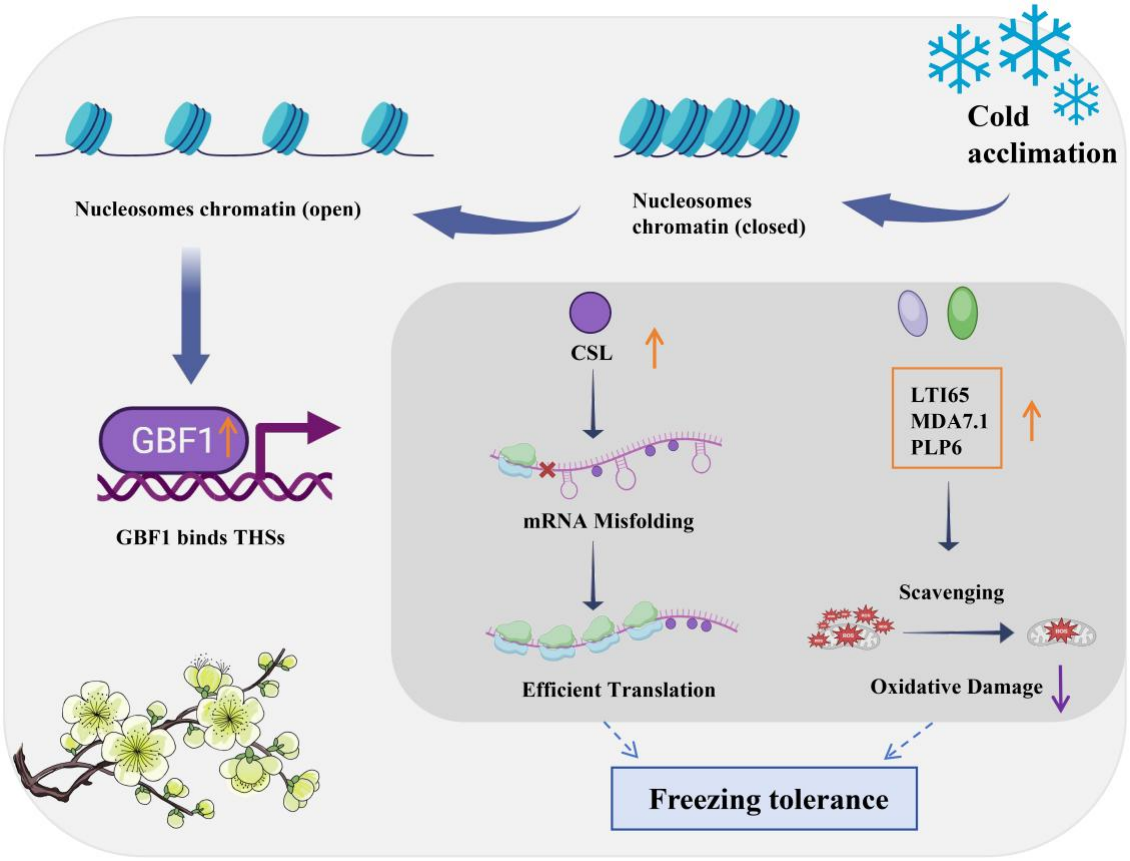
Mechanism of action of the PmGBF1 TF under cold acclimation. THSs, transposase-hypersensitive sites.

When *P. mume* was subjected to low-temperature stress, the cold signal was transmitted to the nuclear temperature response network via the membrane-sensing system. Under cold acclimation conditions, the chromatin structure of the PmGBF1 promoter region tended to be more open, making the site more readily recognized by transcriptional regulatory factors, thereby promoting PmGBF1 transcriptional activation and facilitating its rapid accumulation under low-temperature conditions. As a bZIP TF, PmGBF1 preferentially binds to target genes with open promoters by recognizing the G-box elements in the chromatin-open regions. The activated PmGBF1 played a central regulatory role in the cold response transcriptional network. It coordinated the initiation of a series of transcriptional processes related to cold adaptation by occupying the G-box elements in the promoter regions of multiple target genes. Under cold acclimation, the chromatin of the *PmCSL* and *PmCSLt* promoters opened synchronously and was directly activated by PmGBF1. The CSPs encoded by it buffer low-temperature damage by maintaining RNA homeostasis and translation activity. Meanwhile, PmGBF1 cooperatively regulates genes such as *LTI65*, *MDA7.1*, and *PLP6*, and participates in membrane stability, REDOX homeostasis, and reactive oxygen species clearance (Fig. 6). By coordinating the regulation of multiple genes involved in low-temperature responses, PmGBF1 plays a crucial role in maintaining cellular homeostasis and enhancing overall plant adaptability to low-temperature stress. This result revealed a cold-resistance regulatory model based on chromatin remodeling induced by cold acclimation, with PmGBF1 coordinating hormone signaling and CSP pathways.

## Methods

### Plant materials and growth conditions

The “Zaohua Lve” Mei of *P. mume* were mainly distributed in the Yangtze River–Huai River basin of China. After a long period of intermediate low-temperature acclimatization, they could be grown outdoors in BJ, China, for overwintering at the northernmost limit. The “Zaohua Lve” Mei from BJ and WH were selected as the main plant materials for the experiment. Cuttings of “Zaohua Lve” Mei were collected in BJ and WH during winter, and a grafting operation was performed in spring. Subsequently, the plants were maintained and cultivated under identical conditions. The plants were cultivated under natural environmental conditions, with an average temperature of approximately 22 to 25 °C, a photoperiod of approximately 14 h light/10 h dark, and a relative humidity of 60% to 70%. All low-temperature treatment experiments in this study were conducted before the current-year-grafted plants were about to enter winter dormancy.

### Determination of ion leakage rate and LT_50_ under low-temperature stress conditions

One-year-old branches from WH and BJ's “Zaohua Lve” Mei were used as test materials and placed in a programmable high and low temperature alternating test chamber for low-temperature stress treatment. The temperature gradients were set at 0, −4, −8, −12, −16, and −20 °C, with a cooling rate of 2 °C·h^−1^. Samples were collected after 2 h of constant-temperature treatment at each target temperature, and 3 biological replicates were used for each treatment. The relative ionic permeability was determined by the conductivity method: 0.1 g of fresh tissue was placed in deionized water, and after vacuum permeation treatment, it was shaken at room temperature for 2 h to measure the initial conductivity (EC_1_). Subsequently, the samples were treated in a boiling water bath for 20 min to degrade the cell structure. After cooling to room temperature, the final conductivity (EC_2_) was measured, and the relative ionic permeability was calculated as EC_1_/EC_2_ × 100% (Dexter et al. 1932; Blum and Ebercon 1981). Based on relative ion permeability data from different temperature treatments, a logistic model was fit to the relationship between ion permeability and temperature using the stats package in R. The temperature at which ion permeability reached 50% was defined as the LT_50_ value. This value was used to quantitatively compare the differences in cold resistance of “Zaohua Lve” Mei materials from different sources (Xin and Browse 2000).

### RNA-seq library preparation and data analysis

Based on different cold-acclimation backgrounds, 4 sample groups were established: WH control (WK), WH −4 °C freezing treatment (WF), BA, and BJ post-cold-acclimation −4 °C freezing treatment (BF). Before the freezing treatment, the growth status of all plants was consistent. The freezing treatment was conducted at −4 °C, and samples were collected simultaneously from the control group. The collected samples were immediately frozen in liquid nitrogen and stored at −80 °C for high-throughput sequencing. The tender stem segments (approximately 1 cm) were collected from each treatment group of the “Zaohua Lve” Mei material, with 3 biological replicates per group. Total RNA was extracted using the RNeasy Plant Mini Kit (Qiagen) according to the instructions and further purified using a magnetic scaffold (Invitrogen). Subsequently, a cDNA library was constructed, and high-throughput sequencing was performed on the Illumina HiSeq platform (Bentley et al. 2008) to obtain transcriptome data. The original sequencing data were processed using fastp to remove linker sequences, reads containing >10% unknown bases (N), and reads containing >50% low-quality bases. The clean reads after quality control were aligned to the *P. mume* reference genome using HISAT2 (Kim et al. 2019). Quantitative gene expression and differential expression analyses were performed using DESeq2 (Love et al. 2014). The screening criteria for DEGs are as follows: false discovery rate (FDR) <0.05 and log_2_(fold change) >1. All tests were corrected using multiple hypothesis tests. Genes with a *P*-value < 0.05 were defined as significantly DEGs for subsequent analysis. The public data used in the process were sourced from the Whole Genome Ontology (GO) (Gene Ontology Consortium 2021) and the Kyoto Encyclopedia of Genes and Genomes (KEGG) databases (Kanehisa et al. 2016).

### Construction of ATAC-seq libraries, determination of chromatin accessibility, and integrative analysis

The plant materials and treatment conditions selected for ATAC-seq were the same as those used in the RNA-seq experiment. The ATAC-seq library was optimized according to the method of Buenrostro et al. (2013) and in accordance with the experimental requirements. After library amplification, fragment length distribution and quality were assessed using an Agilent 2100. Qualified libraries underwent 150 bp double-ended sequencing on the Illumina NovaSeq 6000 platform. The quality of the original data was evaluated with FastQC (v0.11.9), and Trimmomatic (Bolger et al. 2014) was then used to remove adapter sequences and low-quality reads. High-quality reads after quality control were aligned to the reference genome of *P. mume* using Bowtie2 (Langmead and Salzberg 2012), and repeated reads, low-quality alignments (MAPQ <30), and mitochondrial-derived reads were further removed to obtain effective alignment data. The peak call was accomplished using MACS3 (Zhang et al. 2008). Subsequently, HTseq (Anders et al. 2015) was used to quantitatively and normalize the peaks of each sample, and the results were input into DESeq2 for differential accessibility analysis. The criteria for identifying differential peaks were set as log_2_(fold change) >1 and FDR <0.1. To systematically analyze the influence of chromatin accessibility changes on gene expression regulation, the differential peaks were mapped to the corresponding genes and integrated with the DEGs identified by RNA-seq for analysis. The number of reads in the promoter region and the distal regulatory region of the gene was converted into RPKM, and the chromatin openness of each gene under different treatment conditions was calculated. According to the established classification method (Meredith et al. 2015), the genes in this study were classified as: “no openness” (RPKM = 0), “high openness” (top 25%), “medium openness” (middle 50%), and “low openness” (bottom 25%).

### Quantitative reverse transcription polymerase chain reaction

Real-time PCR was performed using the TB Green chimeric light method (primers are shown in Table S1). The reaction system and conditions were carried out according to the instructions of the TaKaRaTB Green Premix Ex TaqTM I (Tli RNaseH Plus) kit. Using the *PmPP2A* gene as the internal reference gene, 3 biological replicates and 3 technical replicates were conducted in the experiment, and the relative expression level of the gene was calculated by the 2^−△△Ct^ method.

### Construction of plant overexpression vectors

To verify the biological function of *PmGBF1*, the coding sequence (CDS) was cloned into the pCAMBIA1304 vector containing the hygromycin resistance gene to construct an overexpression vector. The recombinant plasmid was transformed into *A. thaliana* by flower leaching mediated by *Agrobacterium tumefaciens* GV3101. The transforms were screened on MS medium containing 50 mg·L^−1^ hygromycin, and positive strains were identified by PCR. Wild-type and transgenic plants were cultured at 4 °C for 14 d after vulgarization. They were then directly frozen (−5 °C for 1 h) or frozen after cold exercise (4 °C for 3 d; −8 °C for 1 h), respectively. Subsequently, the survival rate and electrical conductivity of the plants were determined, and phenotypic changes were observed.

### Subcellular localization

The CDS fragment of *PmGBF1* was cloned into the pSuper1300-GFP vector (the primer sequence is shown in Table S1) to construct the pSuper1300-*PmGBF1*-GFP fusion expression vector. Subsequently, the fusion vector and the empty control pSuper1300-GFP were transformed into *A. tumefaciens* GV3101. A solution with OD_600_ adjusted to 1.0 was injected into the leaves of *Nicotiana benthamiana*. After 48 h of culturing, the GFP fluorescence signal was observed by laser confocal fluorescence microscopy to analyze the subcellular localization.

### DNA affinity purification sequencing (DAP-seq) technology and data analysis

To further analyze the cold exercise mechanism, DAP-seq (O’Malley et al. 2016) was performed on the PmGBF1 TF. Sequencing was completed on a second-generation sequencing platform, and a PE library (∼300 bp) was constructed for sequencing. Quality control was conducted on the obtained sequencing data. Subsequently, fastp (version 0.23.1) (Chen et al. 2018) software was adopted for data quality control, and the quality information of clean data was statistically analyzed. The clean reads were compared with the plum blossom reference genome using Bowtie 2 (Langmead and Salzberg 2012) with default parameters. The immunoprecipitation call peak was generated using Macs2 (Zhang et al. 2008), and peak calling was performed in narrow peak mode. The coverage depth of all samples was statistically analyzed by bin, standardized to obtain the coverage depth at each position on both sides of the peak-annotated gene TSS, and its distribution was analyzed. The pathway functional enrichment analysis of peak-related genes was performed using KEGG. KEGG analysis was performed on genes with associated peaks located in the promoter–TSS region and enrichment significance ranking in the top 2,000. The analysis criteria are Input_number ≥2 and *P*-value < 0.05.

### Single hybridization of yeast

The promoter fragment of *PmCSL* was cloned into the pAbAi vector and integrated into the YIHGold strain through homologous recombination to generate bait-specific reporter strains. The minimum inhibitory concentrations of each poisoned bait strain were determined on SD/-Ura plates containing different AbA concentrations. The full-length coding region of *PmGBF1* was cloned into the pGADT7 vector as the prey vector. The *PmGBF1*-pGADT7-Rec was transformed into a bait-specific reporter strain, and the prey vector was transformed using the Quick&Easy Yeast Transformation Mix kit. The transformed yeast cells were spread on SD/-Leu/AbA plates and cultured at 30 °C for 3 d (primers were shown in Table S1).

### Statistical analysis

Data were analyzed using variance analysis in SPSS (SPSS 19.0). Data are expressed as mean ± standard deviation. Detailed information on each statistical test is shown in the legend. For comparisons between 2 groups, the Student's *t*-test was used, whereas comparisons among multiple groups were analyzed using one-way analysis of variance. The significance level was set at *P* < 0.05.

## Supplementary Material

kiag333_Supplementary_Data

## Data Availability

The datasets presented in this study can be found in online repositories. The data information is publicly accessible at the National Genomics Data Center under BioProject PRJCA056800.

## References

[kiag333-B1] Anders S, Pyl PT, Huber W. 2015. HTSeq—a Python framework to work with high-throughput sequencing data. Bioinformatics. 31:166–169. 10.1093/bioinformatics/btu638. 25260700 PMC4287950

[kiag333-B2] Artlip TS, Wisniewski ME, Arora R, Norelli JL. 2016. An apple rootstock overexpressing a peach *CBF* gene alters growth and flowering in the scion but does not impact cold hardiness or dormancy. Hortic Res. 3:16006. 10.1038/hortres.2016.6. 26981253 PMC4783695

[kiag333-B3] Bai H et al 2022. *DgbZIP3* interacts with *DgbZIP2* to increase the expression of *DgPOD* for cold stress tolerance in chrysanthemum. Hortic Res. 9:uhac105. 10.1093/hr/uhac105. 35821702 PMC9271009

[kiag333-B4] Baumann K . 2017. Membrane-to-nucleus signals modulate plant cold tolerance. Nat Rev Mol Cell Biol. 18:532. 10.1038/nrm.2017.88. 28377618

[kiag333-B5] Bentley DR et al 2008. Accurate whole human genome sequencing using reversible terminator chemistry. Nature. 456:53–59. 10.1038/nature07517. 18987734 PMC2581791

[kiag333-B6] Blum A, Ebercon A. 1981. Cell membrane stability as a measure of drought and heat tolerance in wheat. Crop Sci. 21:43–47. 10.2135/cropsci1981.0011183X002100010013x.

[kiag333-B7] Bolger AM, Lohse M, Usadel B. 2014. Trimmomatic: a flexible trimmer for Illumina sequence data. Bioinformatics. 30:2114–2120. 10.1093/bioinformatics/btu170. 24695404 PMC4103590

[kiag333-B8] Buenrostro JD, Giresi PG, Zaba LC, Chang HY, Greenleaf WJ. 2013. Transposition of native chromatin for fast and sensitive epigenomic profiling of open chromatin, DNA-binding proteins and nucleosome position. Nat Methods. 10:1213–1218. 10.1038/nmeth.2688. 24097267 PMC3959825

[kiag333-B9] Cai W et al 2018. Overexpression of a wheat (*Triticum aestivum L.*) bZIP transcription factor gene, *TabZIP6*, decreased the freezing tolerance of transgenic *Arabidopsis* seedlings by down-regulating the expression of *CBF*s. Plant Physiol Biochem. 124:100–111. 10.1016/j.plaphy.2018.01.008. 29351891

[kiag333-B10] Cazzolla Gatti R et al 2022. The number of tree species on Earth. Proc Nat Acad Sci U S A. 119:e2115329119. 10.1073/pnas.2115329119. PMC8833151 35101981

[kiag333-B11] Chen S, Zhou Y, Chen Y, Gu J. 2018. Fastp: an ultra-fast all-in-one FASTQ preprocessor. Bioinformatics. 34:i884–i890. 10.1093/bioinformatics/bty560. 30423086 PMC6129281

[kiag333-B12] Chinnusamy V, Zhu J, Zhu JK. 2007. Cold stress regulation of gene expression in plants. Trends Plant Sci. 12:444–451. 10.1016/j.tplants.2007.07.002. 17855156

[kiag333-B13] Consortium TEP . 2012. Chromatin patterns at transcription factor binding sites. Proc Nat Acad Sci U S A. 109:17373–17374. 10.1038/NATURE11247.

[kiag333-B14] Davidson JW et al 2025. Hepatic lipid remodeling in cold exposure uncovers direct regulation of bis(monoacylglycero)phosphate lipids by phospholipase A2 group XV. Cell Metab. 37:1413–1425. 10.1016/j.cmet.2025.04.015. 40373767 PMC12136990

[kiag333-B15] Dexter ST, Tottingham WE, Graber LF. 1932. Investigations of the hardiness of plants by measurement of electrical conductivity. Plant Physiol. 7:63–78. 10.1104/pp.7.1.63. 16652763 PMC439791

[kiag333-B16] Ding Y, Shi Y, Yang S. 2019. Advances and challenges in uncovering cold tolerance regulatory mechanisms in plants. New Phytol. 222:1690–1704. 10.1111/nph.15696. 30664232

[kiag333-B17] Ezer D et al 2017. The G-box transcriptional regulatory code in *Arabidopsis*. Plant Physiol. 175:628–640. 10.1104/pp.17.01086. 28864470 PMC5619884

[kiag333-B18] Gao T et al 2021. Heterologous expression of *Camellia sinensis* late embryogenesis abundant protein gene 1 (*CsLEA1*) confers cold stress tolerance in Escherichia coli and yeast. Hortic Plant J. 7:89–96. 10.1016/j.hpj.2020.09.005.

[kiag333-B19] Gehring M . 2019. Epigenetic dynamics during flowering plant reproduction: evidence for reprogramming? New Phytol. 224:91–96. 10.1111/nph.15856. 31002174 PMC6711810

[kiag333-B20] Gene Ontology Consortium . 2021. The Gene Ontology resource: enriching a GOld mine. Nucleic Acids Res. 49:D325–D334. 10.1093/nar/gkaa1113. 33290552 PMC7779012

[kiag333-B21] Geng Z et al 2025. A U-box E3 ubiquitin ligase *CmPUB15* targets *CmMYB73* to regulate anthocyanin biosynthesis in response to low temperatures in chrysanthemum. New Phytol. 248:1304–1320. 10.1111/nph.70513. 40891718

[kiag333-B22] Gu Y et al 2024. QTL mapping by GWAS and functional analysis of *OsbZIP72* for cold tolerance at rice seedling stage. Crop J. 12:1697–1708. 10.1016/j.cj.2024.07.014.

[kiag333-B23] Guiltinan MJ, Marcotte WR, Quatrano RS. 1990. A plant leucine zipper protein that recognizes an abscisic acid response element. Science. 250:267–271. 10.1126/science.2145628. 2145628

[kiag333-B24] Guo M et al 2024a. Loss of cold tolerance is conferred by absence of the *WRKY34* promoter fragment during tomato evolution. Nat Commun. 15:6667. 10.1038/s41467-024-51036-y. 39107290 PMC11303406

[kiag333-B25] Guo Y et al 2024b. Single-cell RNA sequencing reveals a high-resolution cell atlas of petals in *Prunus mume* at different flowering development stages. Hortic Res. 11:uhae189. 10.1093/hr/uhae189. 39247887 PMC11377181

[kiag333-B26] Hartmann L et al 2015. Crosstalk between two bZIP signaling pathways orchestrates salt-induced metabolic reprogramming in *Arabidopsis* roots. Plant Cell. 27:2244–2266. 10.1105/tpc.15.00163. 26276836 PMC4568499

[kiag333-B27] He Y et al 2025. Binding of PtoRAP2.12 to demethylated and accessible chromatin regions in the PtoGntK promoter stimulates growth of poplar. New Phytol. 245:232–248. 10.1111/nph.20228. 39487606

[kiag333-B28] Hsieh WP, Hsieh HL, Wu SH. 2012. *Arabidopsis* bZIP16 transcription factor integrates light and hormone signaling pathways to regulate early seedling development. Plant Cell. 24:3997–4011. 10.1105/tpc.112.105478. 23104829 PMC3517232

[kiag333-B29] Huang S, Wei P. 2024. Next-generation directed evolution in plants. Mod Agric. 2:e70002. 10.1002/moda.70002.

[kiag333-B30] Huang X et al 2025. Pangenome analysis reveals structural variations associated with citric acid accumulation in *Prunus mume*. Plant Biotechnol J. 23:e70518. 10.1111/pbi.70518. PMC13140591 41440171

[kiag333-B31] Huang Y et al 2023. HSFA1a modulates plant heat stress responses and alters the 3D chromatin organization of enhancer-promoter interactions. Nat Commun. 14:2532. 10.1038/s41467-023-36227-3. 36709329 PMC9884265

[kiag333-B32] Kanehisa M, Sato Y, Kawashima M, Furumichi M, Tanabe M. 2016. KEGG as a reference resource for gene and protein annotation. Nucleic Acids Res. 44:D457–D462. 10.1093/nar/gkv1070. 26476454 PMC4702792

[kiag333-B33] Karlson D, Imai R. 2003. Conservation of the cold shock domain protein family in plants. Plant Physiol. 131:12–15. 10.1104/pp.014472. 12529510 PMC1540277

[kiag333-B34] Kasinathan S, Orsi GA, Zentner GE, Ahmad K, Henikoff S. 2014. High-resolution mapping of transcription factor binding sites on native chromatin. Nat Methods. 11:203–209. 10.1038/nmeth.2766. 24336359 PMC3929178

[kiag333-B35] Kim D, Paggi JM, Park C, Bennett C, Salzberg SL. 2019. Graph-based genome alignment and genotyping with HISAT2 and HISAT-genotype. Nat Biotechnol. 37:907–915. 10.1038/s41587-019-0201-4. 31375807 PMC7605509

[kiag333-B36] Kim JS, Kidokoro S, Yamaguchi-Shinozaki K, Shinozaki K. 2024. Regulatory networks in plant responses to drought and cold stress. Plant Physiol. 195:170–189. 10.1093/plphys/kiae105. 38514098 PMC11060690

[kiag333-B37] Knight MR, Knight H. 2012. Low-temperature perception leading to gene expression and cold tolerance in higher plants. New Phytol. 195:737–751. 10.1111/j.1469-8137.2012.04239.x. 22816520

[kiag333-B38] Langmead B, Salzberg SL. 2012. Fast gapped-read alignment with Bowtie 2. Nat Method. 9:357–359. 10.1038/nmeth.1923. PMC3322381 22388286

[kiag333-B39] Larter M et al 2026. Weak global trade-off between frost and drought resistance in trees. New Phytol. 249:810–828. 10.1111/nph.70718. 41208321 PMC12712442

[kiag333-B40] Li HQ, Li QH, Xing L, Sun GJ, Zhao XL. 2020. Comparison of cold hardiness evaluation of woody species by ELLT and TTCLT. Hortscience. 55:1228–1232. 10.21273/HORTSCI14930-20.

[kiag333-B41] Li N et al 2019. DNA methylation repatterning accompanying hybridization, whole genome doubling and homoeolog exchange in nascent segmental rice allotetraploids. New Phytol. 223:979–992. 10.1111/nph.15820. 30919978

[kiag333-B42] Li P et al 2022a. Genome-wide investigation of the bZIP transcription factor gene family in *Prunus mume*: classification, evolution, expression profile and low-temperature stress responses. Hortic Plant J. 8:230–242. 10.1016/j.hpj.2021.01.009.

[kiag333-B43] Li P et al 2025a. Specific phosphorylation at Ser60 in N terminus of SlCAK1 propels bacterial cold shock protein-induced immunity in Solanaceae plants. Cell Rep. 44:116390. 10.1016/j.celrep.2025.116390. 41066232

[kiag333-B44] Li R et al 2025b. OsbZIP83-OsCOMT15 module confers melatonin-ameliorated cold tolerance in rice. Plant J. 123:e70402. 10.1111/tpj.70402. 40758901

[kiag333-B45] Li Z et al 2022b. The transcription factor bZIP68 negatively regulates cold tolerance in maize. Plant Cell. 34:2833–2851. 10.1093/plcell/koac137. 35543494 PMC9338793

[kiag333-B46] Liu C et al 2018. Early selection of bZIP73 facilitated adaptation of japonica rice to cold climates. Nat Commun. 9:1702. 10.1038/s41467-018-05753-w. 30120236 PMC6098049

[kiag333-B47] Liu C et al 2019. The bZIP73 transcription factor controls rice cold tolerance at the reproductive stage. Plant Biotechnol J. 17:1834–1849. 10.1111/pbi.13104. 30811812 PMC6686130

[kiag333-B48] Liu X, Bulley SM, Varkonyi-Gasic E, Zhong C, Li D. 2023. Kiwifruit bZIP transcription factor AcePosF21 elicits ascorbic acid biosynthesis during cold stress. Plant Physiol. 192:982–999. 10.1093/plphys/kiad121. 36823691 PMC10231468

[kiag333-B49] Love MI, Huber W, Anders S. 2014. Moderated estimation of fold change and dispersion for RNA-seq data with DESeq2. Genome Biol. 15:550. 10.1186/s13059-014-0550-8. 25516281 PMC4302049

[kiag333-B50] Meredith M, Zemmour D, Mathis D, Benoist C. 2015. Aire controls gene expression in the thymic epithelium with ordered stochasticity. Nat Immunol. 16:942–951. 10.1038/ni.3247. 26237550 PMC4632529

[kiag333-B51] Ninkuu V et al 2023. Mitigating biomass recalcitrance for plant-based bioenergy production. Mod Agric. 1:122–141. 10.1002/moda.21.

[kiag333-B52] O’Malley RC et al 2016. Cistrome and epicistrome features shape the regulatory DNA landscape. Cell. 165:1280–1292. 10.1016/j.cell.2016.04.038. 27203113 PMC4907330

[kiag333-B53] Park J et al 2018. Epigenetic switch from repressive to permissive chromatin in response to cold stress. Proc Natl Acad Sci U S A. 115:E5400–E5409. 10.1073/pnas.1721241115. 29784800 PMC6003311

[kiag333-B54] Ren C et al 2021. Characterization of chromatin accessibility and gene expression upon cold stress reveals that the RAV1 transcription factor functions in cold response in *Vitis Amurensis*. Plant Cell Physiol. 62:1615–1629. 10.1093/pcp/pcab115. 34279666 PMC8643690

[kiag333-B55] Ritonga FN, Chen S. 2020. Physiological and molecular mechanism involved in cold stress tolerance in plants. Plants-Basel. 9:560. 10.3390/plants9050560. 32353940 PMC7284489

[kiag333-B56] Sasaki K, Kim MH, Imai R. 2007. *Arabidopsis* COLD SHOCK DOMAIN PROTEIN2 is a RNA chaperone that is regulated by cold and developmental signals. Biochem Biophys Res Commun. 364:633–638. 10.1016/j.bbrc.2007.10.059. 17963727

[kiag333-B57] Shi YT, Ding YL, Yang SH. 2018. Molecular reculation of CBF sicnalinc in colc acclimation. Trends Plant Sci. 23:623–637. 10.1016/j.tplants.2018.04.002. 29735429

[kiag333-B58] Shomo ZD, Li F, Smith CN, Edmonds SR, Roston RL. 2024. From sensing to acclimation: the role of membrane lipid remodeling in plant responses to low temperatures. Plant Physiol. 196:1737–1757. 10.1093/plphys/kiae382. 39028871

[kiag333-B59] Song Y et al 2024. The chromatin remodeller MdRAD5B enhances drought tolerance by coupling MdLHP1-mediated H3K27me3 in apple. Plant Biotechnol J. 22:617–634. 10.1111/pbi.14210. 37874929 PMC10893944

[kiag333-B60] Song ZT, Liu JX, Han JJ. 2021. Chromatin remodeling factors regulate environmental stress responses in plants. J Integr Plant Biol. 63:438–450. 10.1111/jipb.13064. 33421288

[kiag333-B61] Thomashow MF . 1999. Plant cold acclimation: freezing tolerance genes and regulatory mechanisms. Annu Rev Plant Physiol Plant Mol Biol. 50:571–599. 10.1146/annurev.arplant.50.1.571. 15012220

[kiag333-B62] Wang K et al 2025. Horizontally acquired CSP genes contribute to wheat adaptation and improvement. Nat Plants. 11:1465–1475. 10.1038/s41477-025-01952-8. 40148598

[kiag333-B63] Wang P et al 2021. Chromatin accessibility and translational landscapes of tea plants under chilling stress. Hortic Res. 8:143. 10.1038/s41438-021-00529-8. 33931606 PMC8087716

[kiag333-B64] Wang Y et al 2023. Rice chromatin protein OsHMGB1 is involved in phosphate homeostasis and plant growth by affecting chromatin accessibility. New Phytol. 240:727–743. 10.1111/nph.19189. 37553956

[kiag333-B65] Wen S et al 2025. Comprehensive transcriptional regulatory networks in potato through chromatin accessibility and transcriptome under drought and salt stresses. Plant J. 121:70081. 10.1111/tpj.70081. 40086798

[kiag333-B66] Wu Y et al 2022. Genome-wide identification and characterization of the bHLH gene family in an ornamental woody plant *Prunus mume*. Hortic Plant J. 8:531–544. 10.1016/j.hpj.2022.01.004.

[kiag333-B67] Xin Z, Browse J. 2000. Cold comfort farm: the acclimation of plants to freezing temperatures. Plant Cell Environ. 23:893–902. 10.1046/j.1365-3040.2000.00611.x.

[kiag333-B68] Xu Z et al 2023. The bZIP transcription factor SlAREB1 regulates anthocyanin biosynthesis in response to low temperature in tomato. Plant J. 115:205–219. 10.1111/tpj.16224. 36999610

[kiag333-B69] Yang J et al 2020. A lamin-like protein OsNMCP1 regulates drought resistance and root growth through chromatin accessibility modulation by interacting with a chromatin remodeller OsSWI3C in rice. New Phytol. 227:65–83. 10.1111/nph.16518. 32129897

[kiag333-B70] Ye LX et al 2023. A bZIP transcription factor (CiFD) regulates drought- and low-temperature-induced flowering by alternative splicing in citrus. J Integr Plant Biol. 65:674–691. 10.1111/jipb.13390. 36250511

[kiag333-B71] Yen MJ et al 2025. Conserved HSFA1-dependent chromatin dynamics drive heat stress responses in plants. Cell Rep. 44:116714. 10.1016/j.celrep.2025.116714. 41391150

[kiag333-B72] Zhang J et al 2025. Integrated ATAC-seq and RNA-seq reveal *RcHSF30* regulating sHSP and BAG for thermotolerance in rose. Ind Crops Prod. 233:121440. 10.1016/j.indcrop.2025.121440.

[kiag333-B73] Zhang Q et al 2012. The genome of *Prunus mume*. Nat Commun. 3:1318. 10.1038/ncomms2290. 23271652 PMC3535359

[kiag333-B74] Zhang Q et al 2018. The genetic architecture of floral traits in the woody plant *Prunus mume*. Nat Commun. 9:1702. 10.1038/s41467-018-04093-z. 29703940 PMC5923208

[kiag333-B75] Zhang Q, Sun L. 2019. The genome of Prunus mume. Springer. 10.1038/ncomms2290 PMC3535359 23271652

[kiag333-B76] Zhang X, Yu J, Qu G, Chen S. 2024a. The cold-responsive C-repeat binding factors in Betula platyphylla Suk. positively regulate cold tolerance. Plant Sci. 341:112012. 10.1016/j.plantsci.2024.112012. 38311248

[kiag333-B77] Zhang Y et al 2008. Model-based analysis of ChIP-seq (MACS). Methods Mol Biol. 1150:81–95. 10.1186/gb-2008-9-9-r137. 24743991

[kiag333-B78] Zhang Y et al 2024b. A fine-scale *Arabidopsis* chromatin landscape reveals chromatin conformation-associated transcriptional dynamics. Nat Commun. 15:1349. 10.1038/s41467-024-47678-7. 38627396 PMC11021422

[kiag333-B79] Zhao L et al 2026. Heat treatment-induced *PpbZIP43* regulates energy metabolism to enhance postharvest cold tolerance in peach fruit. Postharvest Biol Technol. 233:114032. 10.1016/j.postharvbio.2025.114032.

[kiag333-B80] Zhao W, Neyt P, Van Lijsebettens M, Shen WH, Berr A. 2019. Interactive and noninteractive roles of histone H2B monoubiquitination and H3K36 methylation in the regulation of active gene transcription and control of plant growth and development. New Phytol. 221:1101–1116. 10.1111/nph.15418. 30156703

[kiag333-B81] Zhu JK . 2016. Abiotic stress signaling and responses in plants. Cell. 167:313–324. 10.1016/j.cell.2016.08.029. 27716505 PMC5104190

[kiag333-B82] Zong W et al 2016. Feedback regulation of ABA signaling and biosynthesis by a bZIP transcription factor targets drought-resistance-related genes. Plant Physiol. 171:2810–2825. 10.1104/pp.16.00469. 27325665 PMC4972276

[kiag333-B83] Zou R et al 2025. The roles of epigenetics in the interplay between beneficial rhizobacteria and plants. Mod Agric. 3:e70017. 10.1002/moda.70017.

