# Supplementary material for: Molecular mechanism of cold acclimation regulating freezing tolerance in Prunus mume
Source: Plant Physiol. 2026 Jun 3;201(2):kiag333. doi: 10.1093/plphys/kiag333 (PMC13316940; doi:10.1093/plphys/kiag333)
Supplement: kiag333_Supplementary_Data [file kiag333_supplementary_data.zip › Supplementary Figure.docx]

Supporting Information


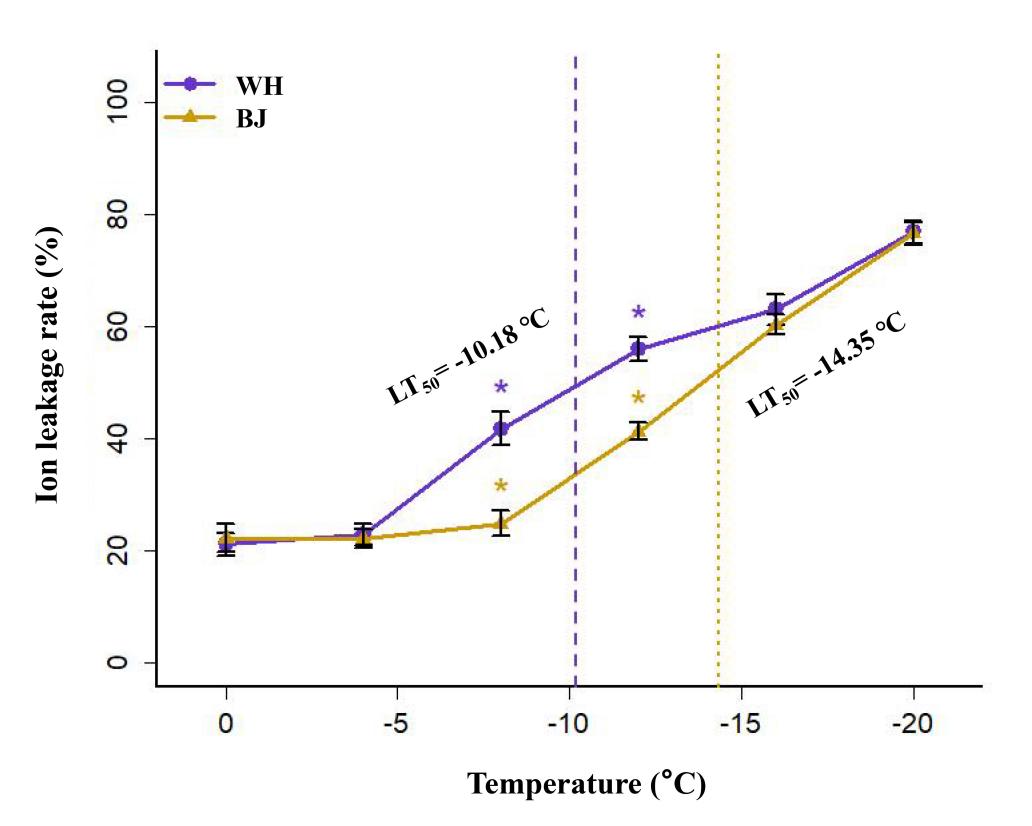


**Figure S1.** The influence of different low-temperature stresses on the ion leakage rate of two *Prunus mume*.The dotted line represents the median death temperature (LT_50_) calculated by Logistic fitting. The data is expressed as the mean ± standard error (SE) (n = 3), and * indicates a significant difference between the two materials under the same temperature treatment (P < 0.05).WH: Wuhan, BJ: Beijing.


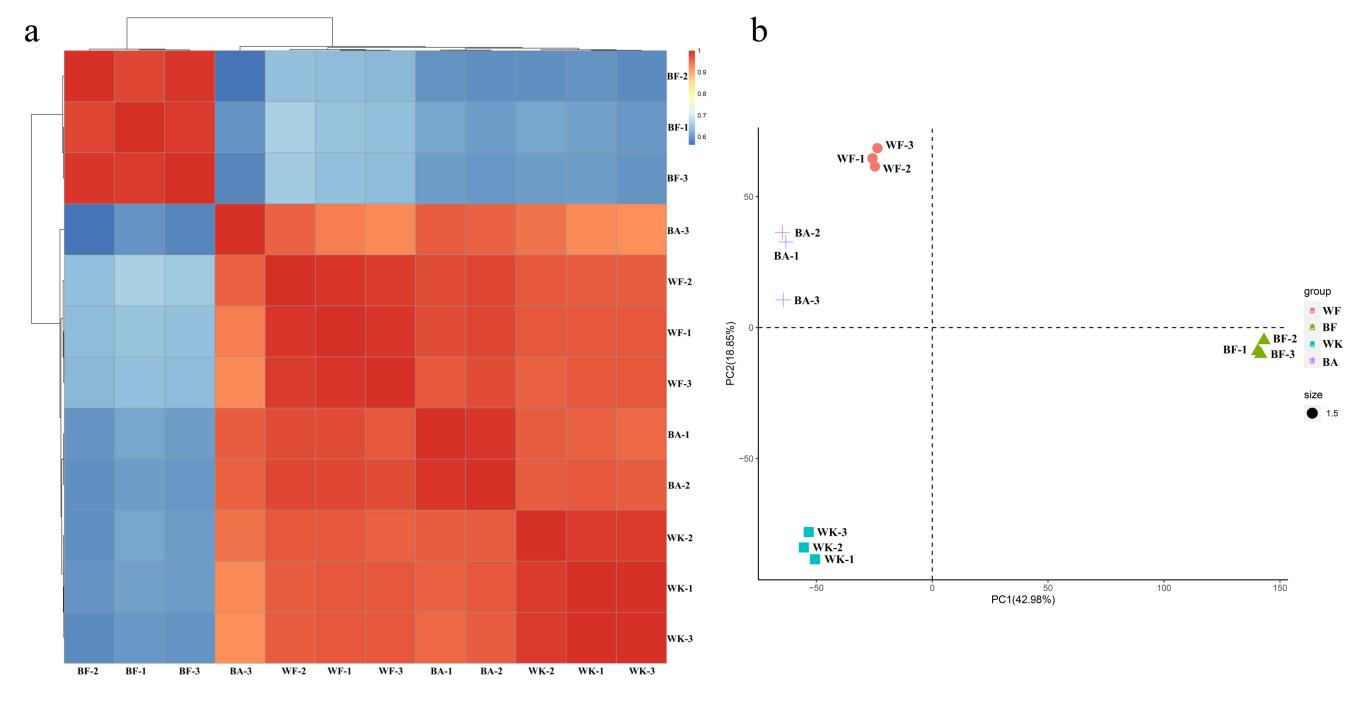


**Figure S2.** Sample Correlation Analysis. **(**a) Heat map of inter-sample correlation. (b) PCA clustering graph between samples.


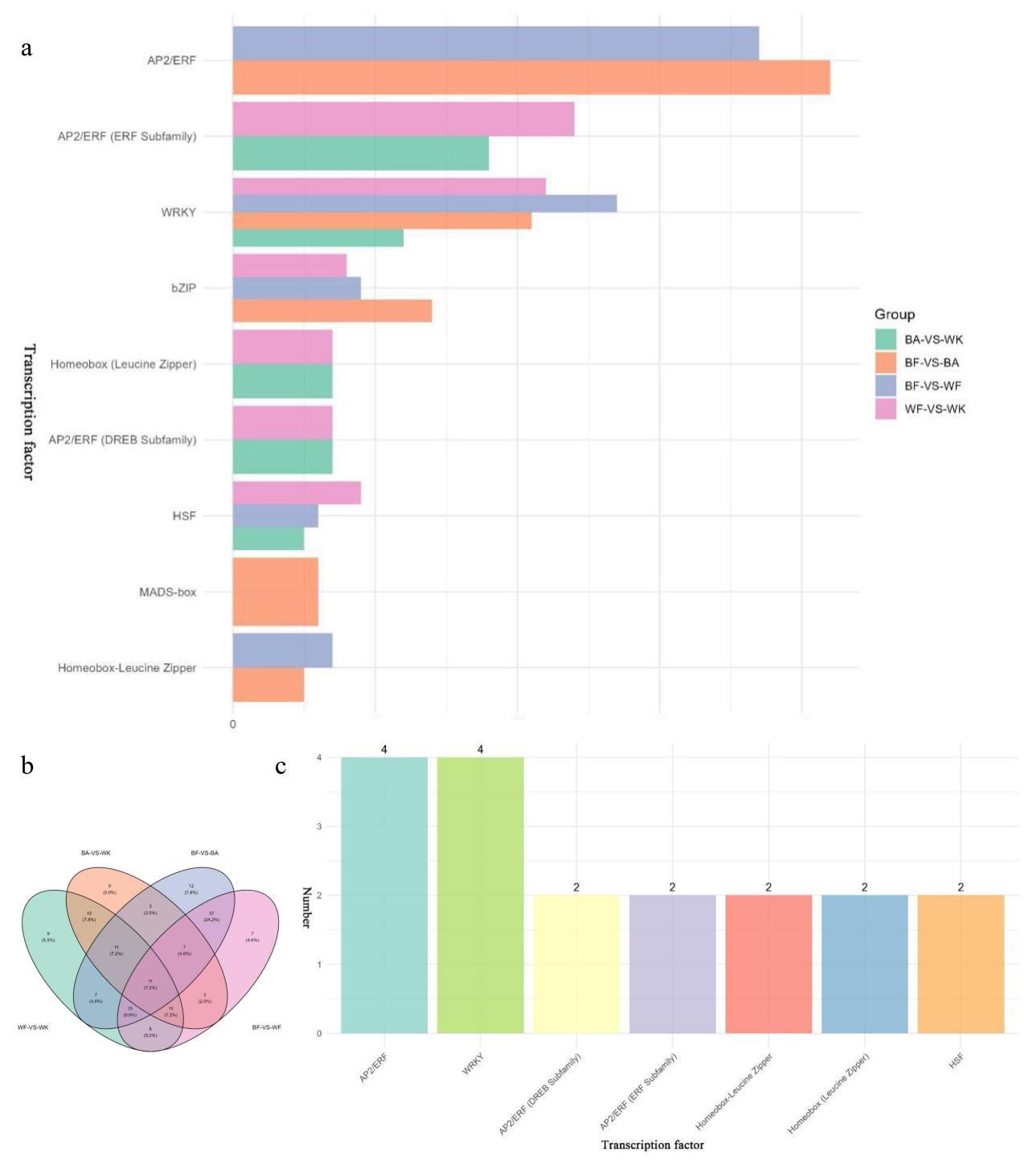


**Figure S3.** Comparative analysis of transcription factor family composition among groups. **(**a) Distribution of the top five transcription factor families in each group. (b) Overlap of transcription factors among the four groups illustrated by a Venn diagram;c Family distribution of transcription factors shared among the groups.


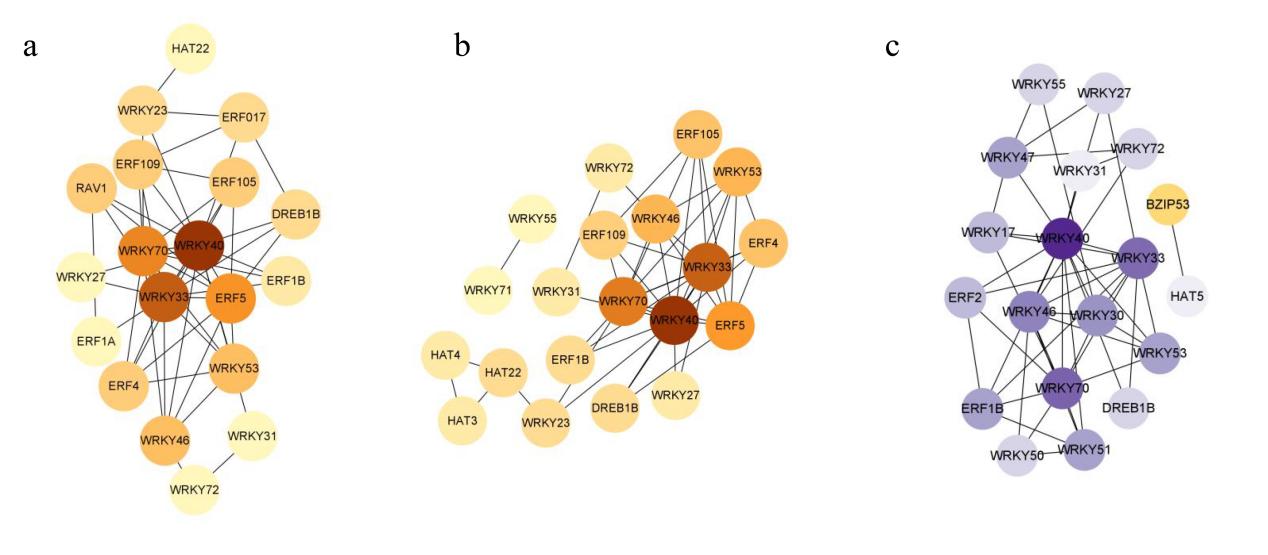


**Figure S4.** Protein–protein interaction (PPI) networks of transcription factors under different comparisons. **(**a) PPI network of transcription factors in the BA vs WK comparison. (b) PPI network of transcription factors in the WF vs WK comparison. (c) PPI network of transcription factors in the BF vs WF comparison. Top 20 nodes ranked by connectivity; orange and purple indicate up- and down-regulation, respectively.


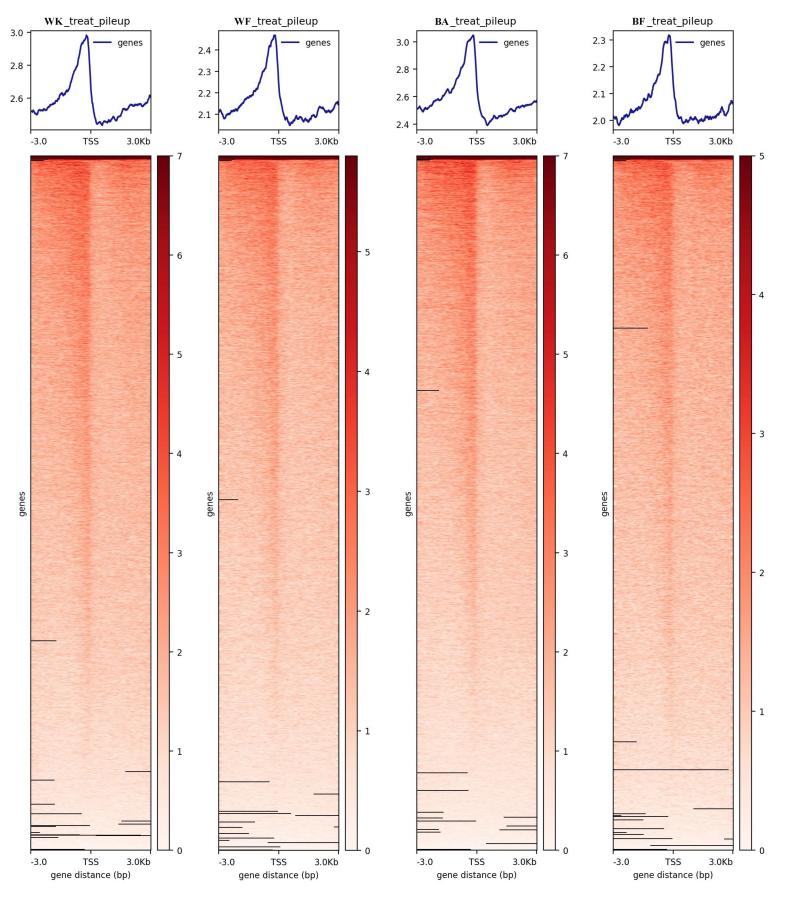


**Figure S5.** Signal enrichment around the transcription start site (TSS).


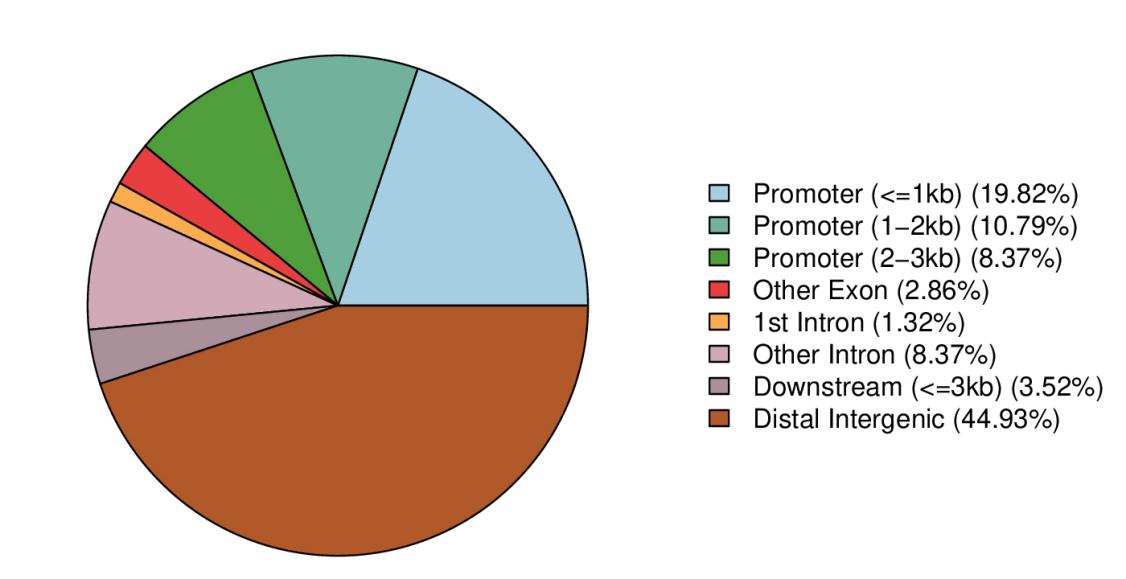


**Figure S6.** Genomic distribution of ATAC-seq peaks


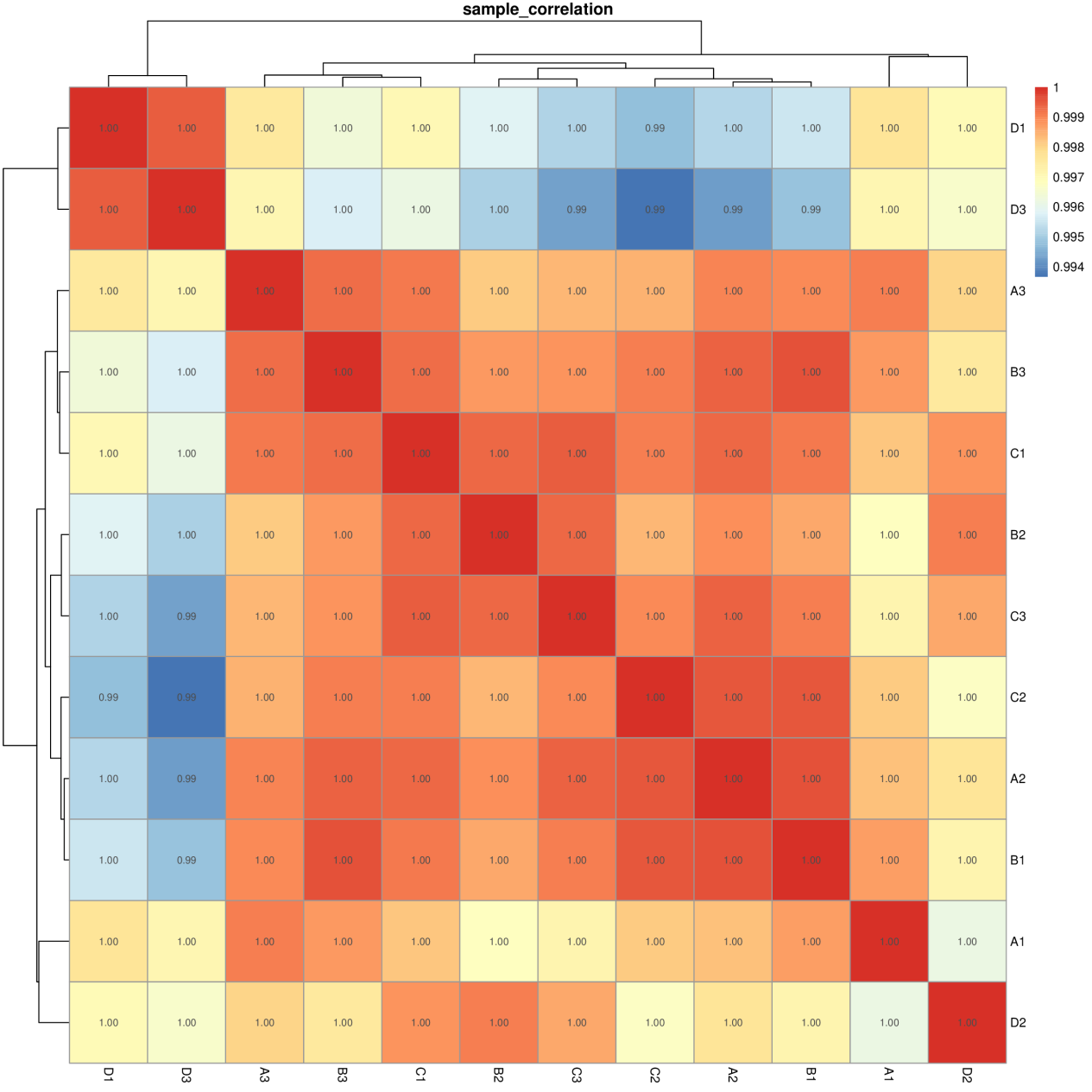


**Figure S7.** llustration of inter-sample correlation (Based on FPKM). A: WK, Wuhan control; B: WF, freezing treatment in Wuhan; C: BA, Beijing control; D: BF, freezing treatment in Beijing.


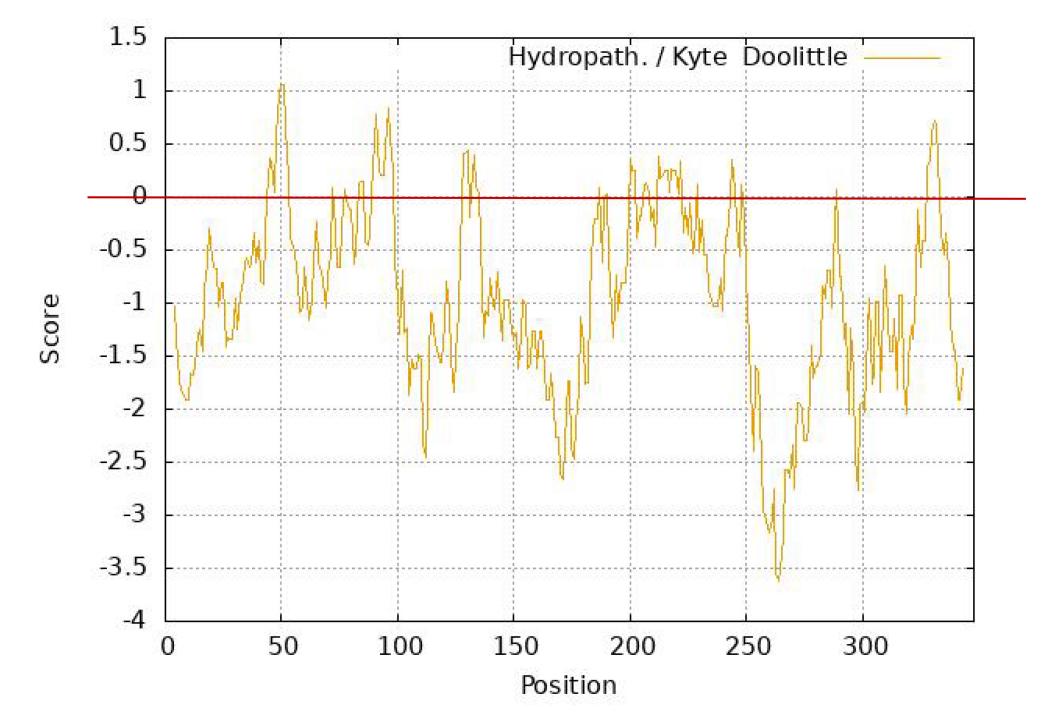


**Figure S8.** Analysis of the hydrophilicity and hydrophobicity of GBF1 protein.


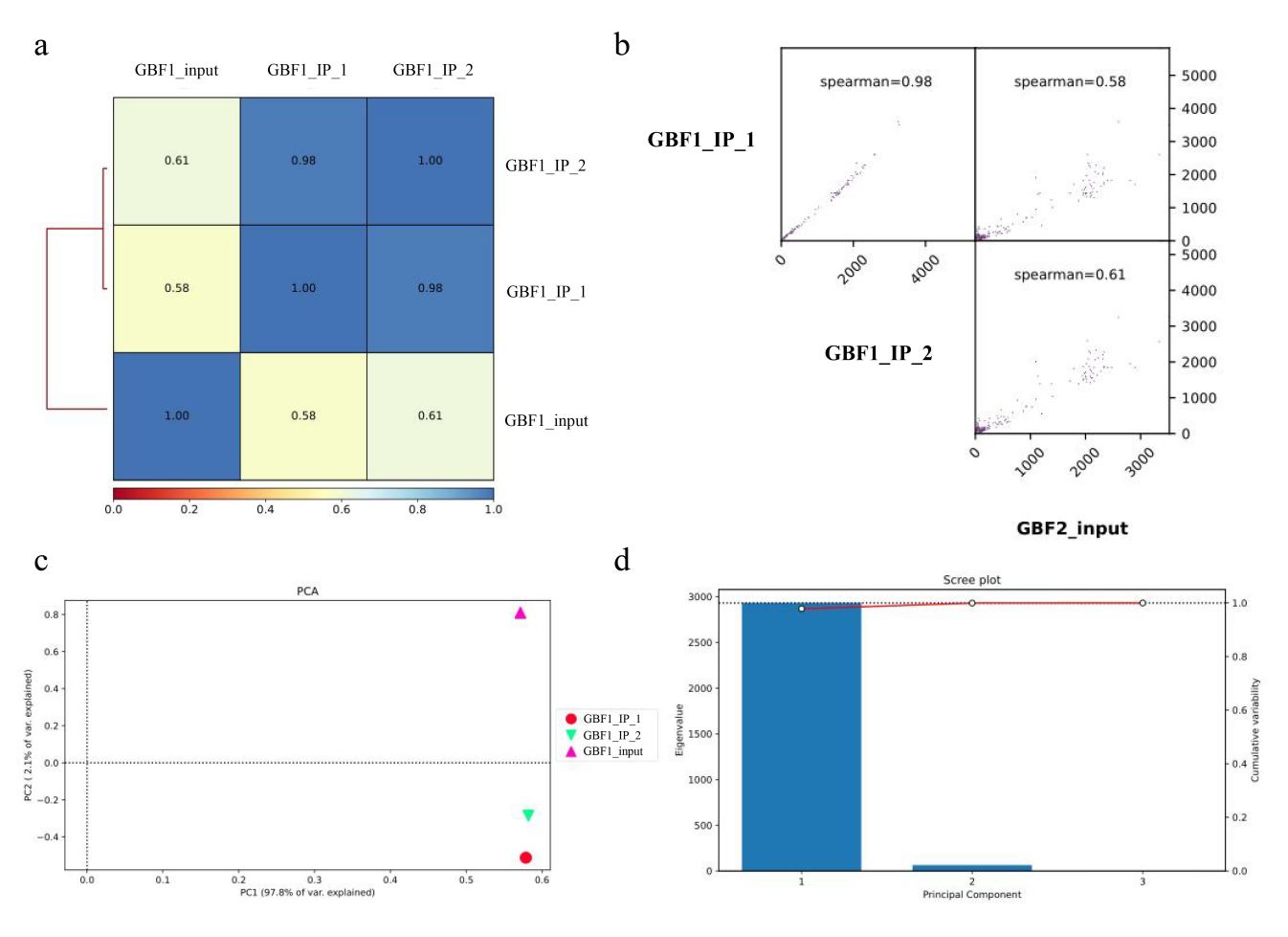


**Figure S9.** Detection of biological repeat correlation of samples. (a) Hierarchical clustering heat map between samples. (b) Scatter plot of TPM correlation within each bin interval between samples. (c) Principal component analysis clustering graph of the sample; d Bar charts of the variations of each principal component


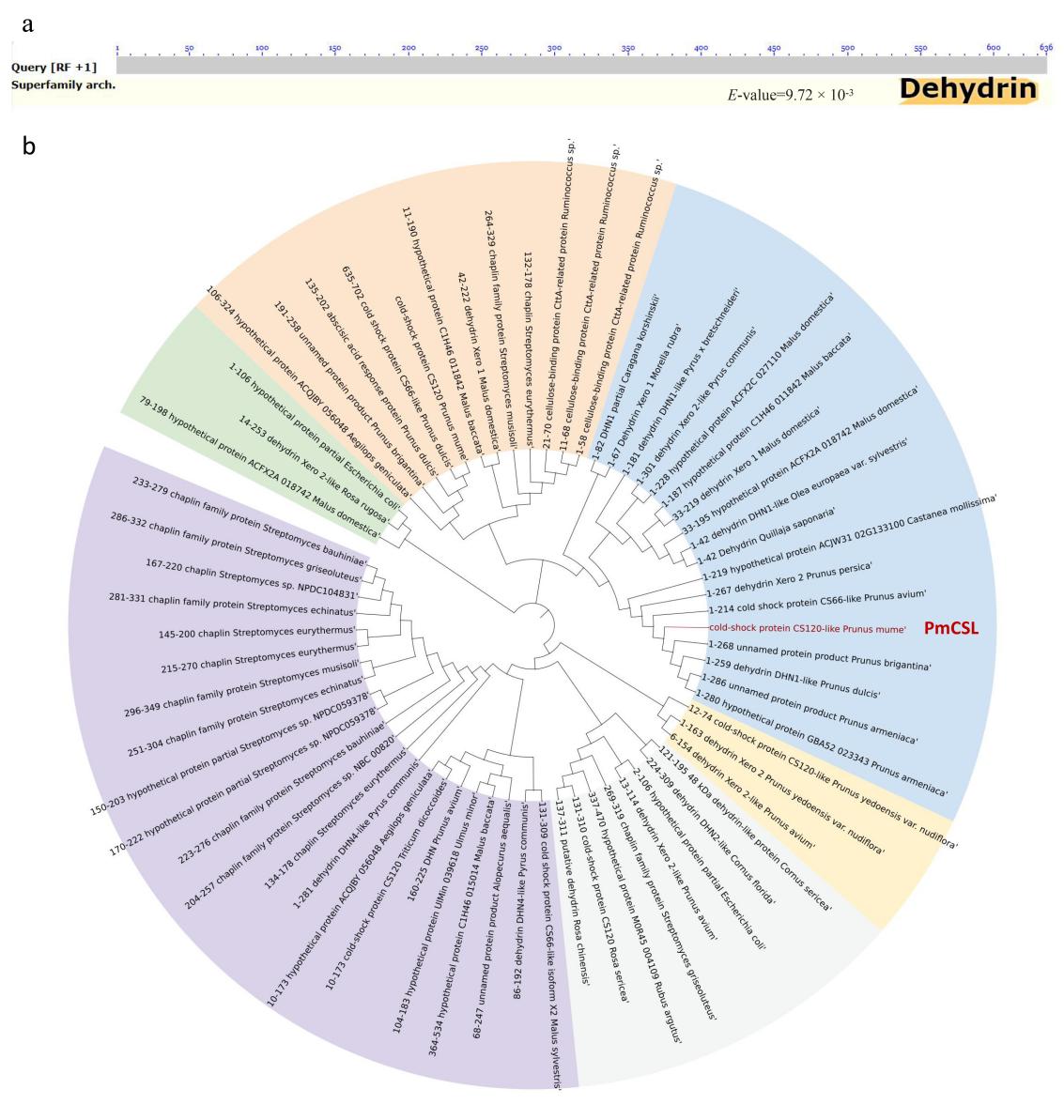


**Figure S10.** Structural domain and phylogenetic analysis of PmCSL. (a) Analysis of the protein domain of PmCSL. (b) Analysis of the evolutionary tree of the PmCSL system.
